# Room-temperature dynamic nuclear polarization enhanced NMR spectroscopy of small biological molecules in water

**DOI:** 10.1038/s41467-021-27067-0

**Published:** 2021-11-25

**Authors:** Danhua Dai, Xianwei Wang, Yiwei Liu, Xiao-Liang Yang, Clemens Glaubitz, Vasyl Denysenkov, Xiao He, Thomas Prisner, Jiafei Mao

**Affiliations:** 1 Institute of Physical and Theoretical Chemistry, Goethe University Frankfurt, 60438 Frankfurt am Main, Germany; 2 Center for Biomolecular Magnetic Resonance, Goethe University Frankfurt, 60438 Frankfurt am Main, Germany; 3 Shanghai Engineering Research Center of Molecular Therapeutics and New Drug Development, School of Chemistry and Molecular Engineering, East China Normal University, Shanghai, 200062 China; 4 College of Science, Zhejiang University of Technology, Hangzhou, Zhejiang 310023 China; 5 Jiangsu Key Laboratory of Advanced Organic Materials, School of Chemistry and Chemical Engineering, Nanjing University, Nanjing, 210023 China; 6 State Key Laboratory of Coordination Chemistry, School of Chemistry and Chemical Engineering, Nanjing University, Nanjing, 210023 China; 7 Institute of Biophysical Chemistry, Goethe University Frankfurt, 60438 Frankfurt am Main, Germany; 8 NYU-ECNU Center for Computational Chemistry at NYU Shanghai, Shanghai, 200062 China

**Keywords:** Biophysical chemistry, Analytical chemistry, Theoretical chemistry

## Abstract

Nuclear magnetic resonance (NMR) spectroscopy is a powerful and popular technique for probing the molecular structures, dynamics and chemical properties. However the conventional NMR spectroscopy is bottlenecked by its low sensitivity. Dynamic nuclear polarization (DNP) boosts NMR sensitivity by orders of magnitude and resolves this limitation. In liquid-state this revolutionizing technique has been restricted to a few specific non-biological model molecules in organic solvents. Here we show that the carbon polarization in small biological molecules, including carbohydrates and amino acids, can be enhanced sizably by in situ Overhauser DNP (ODNP) in water at room temperature and at high magnetic field. An observed connection between ODNP ^13^C enhancement factor and paramagnetic ^13^C NMR shift has led to the exploration of biologically relevant heterocyclic compound indole. The QM/MM MD simulation underscores the dynamics of intermolecular hydrogen bonds as the driving force for the scalar ODNP in a long-living radical-substrate complex. Our work reconciles results obtained by DNP spectroscopy, paramagnetic NMR and computational chemistry and provides new mechanistic insights into the high-field scalar ODNP.

## Abstract (web summary)

Dynamic nuclear polarization (DNP) greatly improves the NMR sensitivity, but its implementation in aqueous solutions is challenging. Here the authors demonstrate carbon polarization enhancement via in situ Overhauser DNP in small biomolecules in water at room temperature and high magnetic field.

## Introduction

Nuclear magnetic resonance (NMR) spectroscopy is an indispensable powerful technique for probing the molecular structure, dynamics, and chemical properties at or even beyond the atomic resolution. It has strong impacts on a broad range of applications in chemistry, biology, material science, environmental science as well as chemical and pharmaceutical industries. However, all these applications are limited by the low inherent sensitivity of this technique. Since the early days of NMR spectroscopy, the demands for higher sensitivity have been driving the developments in instrumentation, methodology, and the understanding of NMR spin dynamics^1^. One of the popular approaches for boosting NMR sensitivity is dynamic nuclear polarization (DNP), in which nuclear spins are hyperpolarized via the polarization transfer from unpaired electron spins under microwave irradiation^2–9^. In most DNP schemes, the unpaired electron spins are introduced as paramagnetic radicals or metal centers. Since the electron spin has a much higher gyromagnetic ratio than any nuclear spins, that is −658-fold of ^1^H nucleus and −2617-fold of ^13^C nucleus, tremendous sensitivity enhancements can be anticipated. Such a remarkable potential has sparked enthusiasms on developing DNP techniques for numerous NMR applications^5,10–27^.

The past decade has already witnessed the transformation of solid-state NMR (ssNMR) spectroscopy by DNP^2,5,6,9^. Solid-state DNP experiments are carried out mostly at cryogenic temperatures (usually below 120 K), at which the electron spin relaxation times are long enough for the efficient microwave saturation in solids. Such low operation temperatures quench most molecular dynamics, prohibit chemical processes, and in many cases compromise the spectral resolution. An alternative approach, namely the dissolution DNP, combines the low-temperature solid-state DNP and the room-temperature liquid-state NMR via a rapid dissolution/melting step. However, it is a single-shot experiment and a freeze-thaw procedure has to be applied to the samples^17,28–32^. Different from all the abovementioned approaches, the in situ liquid-state Overhauser DNP (ODNP) process permits to hyperpolarize the target molecules directly in solution at room-temperature^4,10,12,13,16,18,20,22,23,25,33^.

In liquid-state DNP the electron-nucleus (e-N) polarization transfer is mediated by the electron-nucleus Overhauser effect (OE) driven by molecular motions. The physical principle of OE is the e-N cross-relaxation caused by dynamic fluctuations of the e-N spin−spin interactions. Unpaired electron spins interact with nuclear spins via dipolar interaction and scalar hyperfine interactions, both of which can contribute to the ODNP. The ODNP efficiency relies on both the magnitude and the dynamics of the e-N interactions. A more detailed introduction on the theoretical background of ODNP can be found in Supplementary Note 1. At high magnetic fields (e.g., 9.4 T), molecular motions at the sub-ps time scale, or in the THz frequency regime, are required for efficient ODNP. In aqueous solutions of nitroxide radicals, translational and rotational motions, which drive the stochastic e-N dipolar crosstalk upon the radical-water encounter events, occur within this time scale. These motions lead to a remarkable negative ODNP-enhancement of the water ^1^H NMR signal at 9.4 T^34^. However, such molecular motions become significantly retarded upon increasing the molecular sizes. Consequentially the efficiency of ODNP driven by dipolar interactions drops significantly on more complex molecules^35^.

Distinct from the dipolar ODNP, the scalar ODNP depends on the fluctuation of the e-N Fermi contact interactions. The *chemical* interaction between the radical and the substrate molecule permits the dispersion of spin density from the radical to the specific nuclear positions in the substrate molecule. This scalar e-N Fermi contact fluctuates upon the radical-substrate chemical encounter-disassociation events and/or during the structural rearrangement of the radical-substrate complex in solution^20,25,36,37^. The recently observed tremendous positive liquid-state ODNP enhancements on ^13^C signals of various organic compounds show the great potential of this mechanism at high magnetic field^20,25^. However, these work has been limited on a few non-biological model compounds so far, for example, halogenated hydrocarbons, diketones, and benzene derivatives^19,20,25^. Moreover, all the previous studies have been restricted to organic solvents with lower microwave absorption at high magnetic field in order to reduce the sample heating by microwave, a major challenge for high-field room-temperature ODNP. Here, we have successfully polarized a number of structurally and chemically diverse small biological molecules in water directly at room-temperature and at high magnetic field (9.4 T, 263 GHz electron Larmor frequency). Motivated by the observed trend between scalar ODNP enhancement and paramagnetic NMR shift, we have also expanded our ODNP target list to biologically relevant heterocyclic compound indole. Extensive density functional theory (DFT) calculations and quantum mechanics/molecular mechanics molecular dynamics (QM/MM MD) simulations have provided new mechanistic insights into scalar ODNP in various small biological molecules as well as in a long-living H-bonded radical-substrate complex.

## Results

### Room-temperature ODNP ^13^C NMR of imidazole in water

**Fig. 1:**
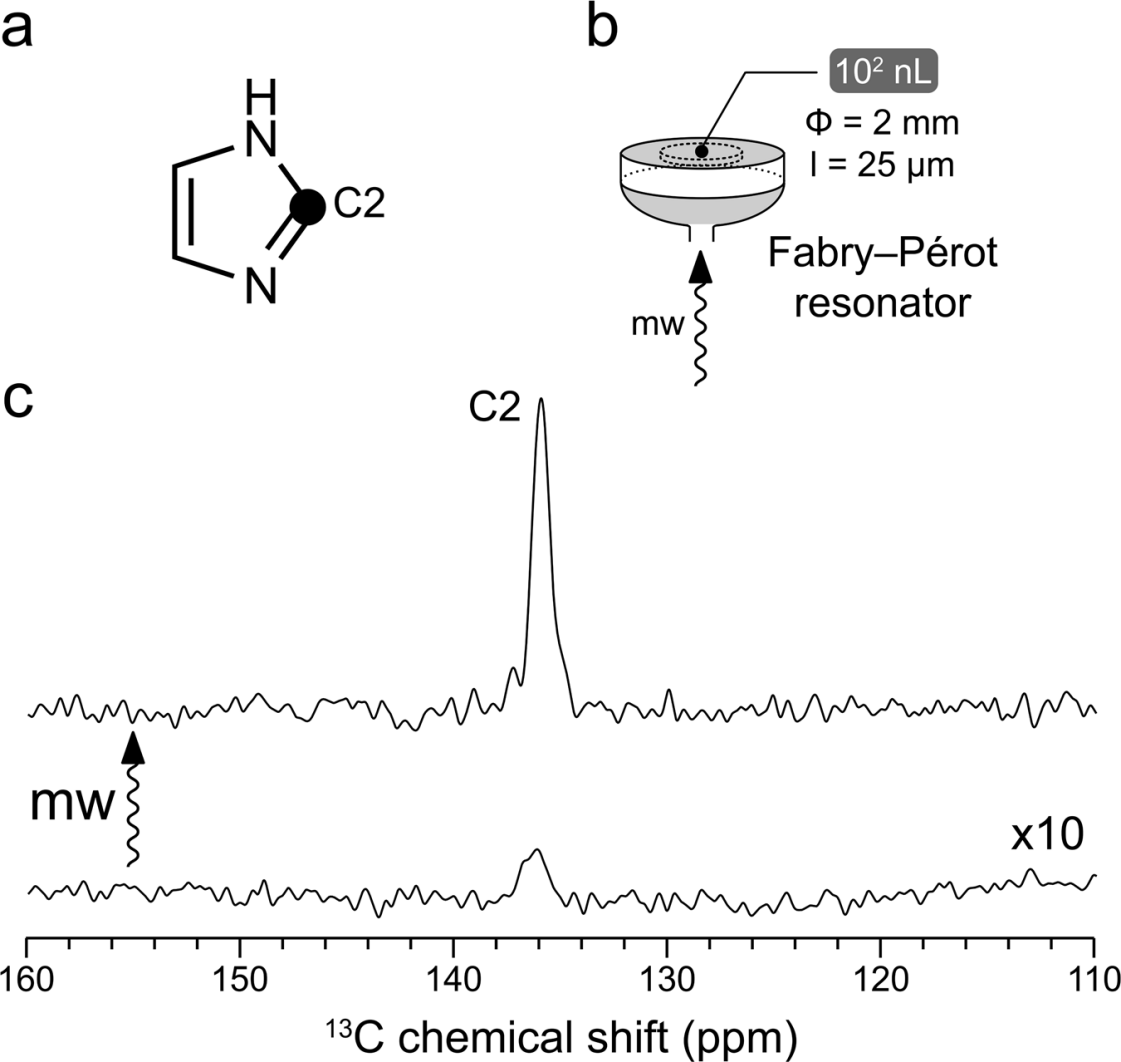
Room-temperature ODNP-enhanced ^13^C NMR of ^13^C2 imidazole in D_2_O at 9.4 T. **a** The chemical structure of imidazole molecule. The black circle indicates ^13^C labeled position. **b** Scheme of Fabry-Pérot resonator^16,39^. The sample (10^2^ nL) has been laid as a thin layer on the flat mirror (gray). The sample diameter (Φ) and thickness (*l*) are shown. The microwave beam (shown as mw arrow) enters the resonator via a hole in the middle of a spherical mirror (gray). **c** Spectra obtained with (up) and without (down) microwave (mw) are presented. A 50-fold ODNP enhancement on ^13^C signal has been obtained. TEMPOL (100 mM) was used as the hyperpolarizing agent. The spectra are scaled with number of scans for visualizing directly the enhancement factor. The microwave-off spectra are further scaled up by 10-fold for the better visualization.

The sample heating under microwave irradiation poses one major challenge on room-temperature liquid-state ODNP spectroscopy. In some previous work, this problem was alleviated partially by restricting the experiments to organic solvents of low polarity^19,20,25^. However, on the aqueous samples relevant to biological applications, the microwave heat deposition becomes inevitably more severe (loss tangent of water tanδ = 1.05 at 20 °C and 0.26 THz)^38^. For tackling this issue, we have chosen a home-built liquid-state DNP instrumentation platform based on a Fabry–Pérot microwave double resonance structure (Fig. 1b)^39^ that permits efficient excitation of the radical electron spin with an excellent temperature management. In the Fabry–Pérot microwave resonator, a highest microwave B-field strength is reached at the sample position, which is required for the microwave saturation of the radical electron spin transition in DNP experiments. At the same time, the microwave electric field component, which leads to sample heating, is near zero on the metal surface where the thin flatdisc-like sample (10^1^ μm thickness, Fig. 1b) is located. In addition, since the samples are situated directly onto a heat-conducting flat metal mirror (Fig. 1b), the heat dissipation from the sample to its environment is rather efficient. Previously this design has already been successfully used for dipolar ODNP ^1^H NMR experiments on water and on hydrated lipid bilayers^16,39^.

Although our setting reduces substantially the sample heating, it remains rather technically challenging to perform the ODNP NMR experiments. Here, instead of the blind “trial-and-error” search for molecules with large scalar ODNP enhancements, we have first sought a potential indicator of the scalar ODNP performance. The scalar ODNP relies on the Fermi contact mediated by the chemical interactions between radical and substrate molecules. The Fermi contact leads to three distinct but closely related spectroscopic consequences, namely the signal enhancement in scalar ODNP NMR spectroscopy, the hyperfine pattern in EPR spectroscopy, and the well-known Fermi contact shift in paramagnetic NMR spectroscopy. The hyperfine interactions in radical-substrate complexes have been explored extensively by EPR spectroscopy and computational chemistry. Indeed it has been shown that the hyperfine constant, which quantifies the magnitude of the Fermi contact, is correlated with the scalar ODNP enhancement^40^. In this work, inspired originally by a Google search (Supplementary Note 6), we have recalled the link between paramagnetic NMR and ODNP spectroscopy. As the contact shift also reports the magnitude of the Fermi contact, it could be taken presumably as an NMR indicator for the ODNP performance similar to the hyperfine coupling constant explored in the previous work^40^. In particular, the molecules that do not interact chemically with radicals should show little paramagnetic NMR shift. Such molecules could be excluded efficiently prior to the technically challenging ODNP experiments, which improves the winning rate of our initial search for promising scalar ODNP targets.

The Fermi contact shift has been one of the major topics in paramagnetic NMR spectroscopy over many decades^41–45^. We have revisited the rich collection of literatures covering this topic and have identified a series of paramagnetic NMR studies, in which a wide range of radical-interacting molecules have been reported^46–53^. Interestingly some halogenated compounds showing large ODNP enhancements as reported recently^20,25^ also exhibit large ^13^C paramagnetic NMR shifts^54^. In this work, we have taken the molar-free ^13^C paramagnetic NMR shifts ($\overline{{\delta }_{{{{{{\rm{para}}}}}}}}$) for the comparison of various compounds, which normalize the scale of the paramagnetic NMR shift over the radical concentration. Within these paramagnetic NMR literatures, a water-soluble heterocyclic compound imidazole, which is the sidechain moiety of the amino acid histidine, is reported to interact with TEMPO-type radicals via an intermolecular H-bond and exhibits some of the most remarkable carbon $\overline{{\delta }_{{{{{{\rm{para}}}}}}}}$^53^. We have therefore selected this compound as a candidate for ODNP ^13^C NMR experiments in water on our high-field liquid-state DNP setup.

The solution of ^13^C2-imidazole (Fig. 1a) in D_2_O has been loaded as a thin layer into the Fabry–Pérot microwave resonator (Fig. 1b). The water-soluble radical TEMPOL (100 mM) has been used as the ODNP polarization agent. Pleasingly a large ODNP ^13^C enhancement of 50 has been obtained with this sample (Fig. 1c and Supplementary Table 3). This finding has encouraged us to explore other families of water-soluble molecules for ODNP experiments.

### Room-temperature ODNP ^13^C NMR of small biological molecules in water

**Fig. 2:**
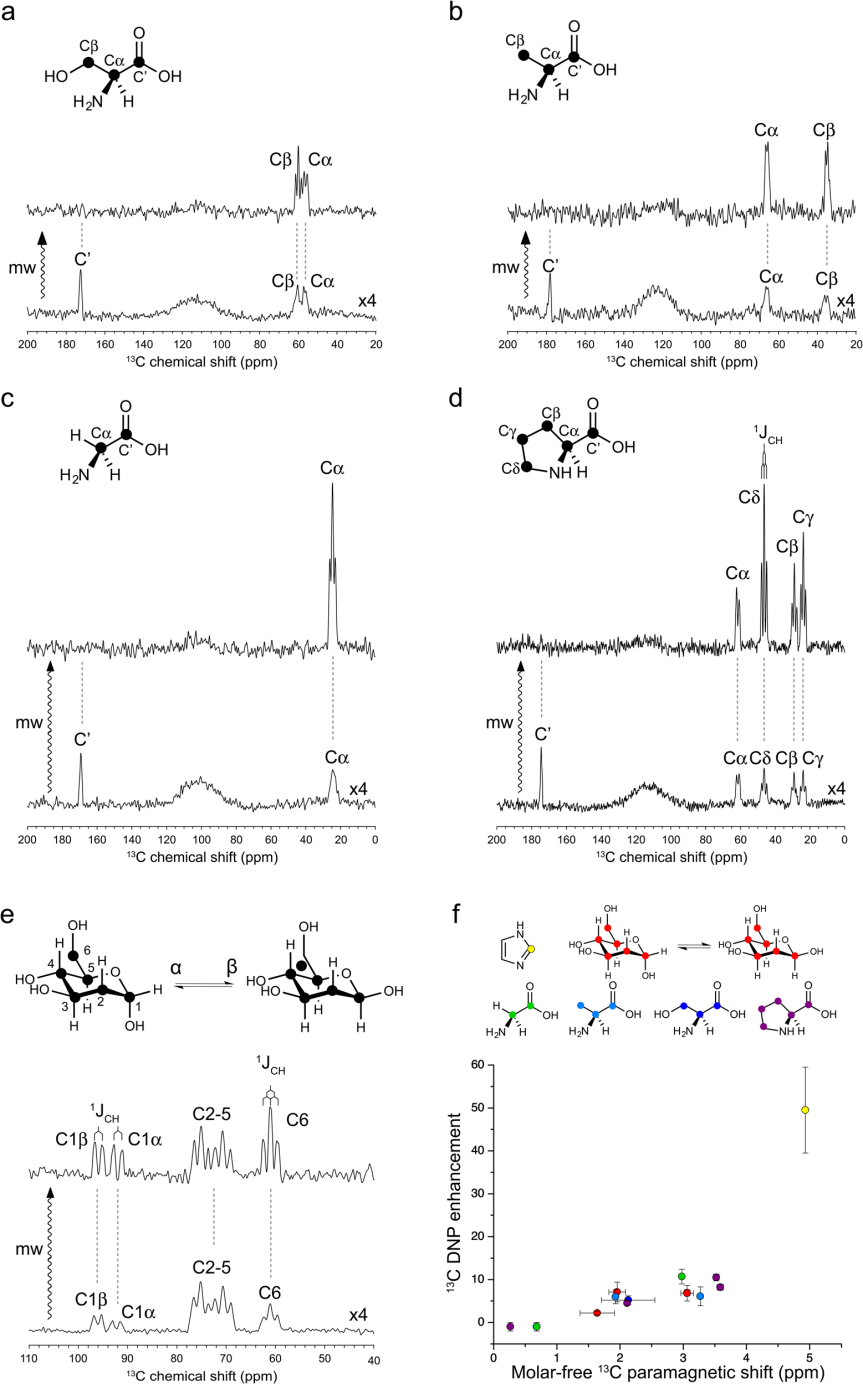
Room-temperature ODNP NMR of small biological molecules in H_2_O at 9.4 T. TEMPOL (100 mM) was used as the hyperpolarizing agent. The ODNP-enhanced ^13^C NMR spectra of U–^13^C serine (**a**), alanine (**b**), glycine (**c**), proline (**d**), and glucose (**e**) are scaled with the number of scans for visualizing directly the enhancement factor. All the microwave (mw)-off spectra in (**a**–**e**) are further scaled up by four-fold for the better visualization. The resolved ^1^J_CH_ are showcased in spectral (**d**, **e**). **f** A positive trend of ODNP ^13^C enhancement over molar-free paramagnetic ^13^C NMR shift $\overline{{\delta }_{{{{{{\rm{para}}}}}}}}$ is visualized. The data on various molecules are presented in colors. The error of $\overline{{\delta }_{{{{{{\rm{para}}}}}}}}$ is defined as fitting error or standard deviation of $\overline{{\delta }_{{{{{{\rm{para}}}}}}}}$ values on carbons showing overlapping signals on ODNP spectra. The error of ODNP enhancement is determined from signal-to-noise ratio. More details about the error calculations can be found in “Methods”. Source data are provided as a Source Data file.

Encouraged by the initial success on imidazole, we moved on to structurally more complex small biological molecules. In this work, we have focused on carbohydrate and amino acids (Fig. 2) bearing amine and/or hydroxyl groups that could serve potentially as H-bond donor to TEMPO-type radicals. In particular, we have targeted the highly water-soluble glucose (Fig. 2e) and amino acids (Fig. 2a–d, glycine, serine, alanine and proline). As indicated by the significant carbon $\overline{{\delta }_{{{{{\rm{para}}}}}}}$ (Supplementary Fig. 2 and Supplementary Table 4), all these small biological molecules interact chemically with TEMPOL in water. We have further subjected all these compounds to ODNP ^13^C NMR experiments in H_2_O using the same instrumentation setting for imidazole. Sizable ODNP ^13^C enhancements have been obtained on all these molecules (Fig. 2a–e). Notably, our spectral resolution even permits to resolve the signal splitting by ^1^J_CH_ couplings (Fig. 2a–e and Supplementary Table 3), which significantly outperforms the DNP ssNMR spectroscopy on a frozen solution sample at the same magnetic field (Supplementary Fig. 1d).

The excellent spectral resolution also permitted to detect the site-specific ODNP ^13^C enhancement for most of the carbons in these small biological molecules. The chemical shift assignment of the ODNP-enhanced ^13^C spectra of these molecules can be found in Fig. 2a–e. The corresponding site-specific ODNP enhancement factors are summarized in Supplementary Table 3 and Fig. 2f. Up to 11-fold ODNP enhancement of ^13^C signals have been obtained on these small biological molecules. The site-specific ODNP enhancement has been observed on all these molecules. As expected, a qualitative positive trend has been observed between the carbon $\overline{{\delta }_{{{{{{\rm{para}}}}}}}}$ and the ODNP enhancement factor within and across the tested water-soluble molecules (Fig. 2f and Supplementary Table 4).

In glucose, the C1 and C6 carbons show larger ODNP enhancements than the other carbons (Fig. 2e and Supplementary Table 3). These two carbons are less sterically hindered than other carbons and are therefore more likely accessible by TEMPOL radical as also indicated by their larger $\overline{{\delta }_{{{{{{\rm{para}}}}}}}}$. In the four tested amino acids, the carboxylate carbons all show negative ODNP enhancements (Fig. 2a–d and Supplementary Table 3), which evidences the dominating role of dipolar ODNP mechanism on these carbons similar to that found on carbonyl and ester carbons in various organic compounds^25^. As indicated by their small $\overline{{\delta }_{{{{{{\rm{para}}}}}}}}$ (Supplementary Table 4), these carboxylate groups are not involved in the direct interactions with TEMPOL radical. In contrast, the aliphatic carbons in amino acids and glucose all show positive ODNP enhancements, which supports the prevailing scalar ODNP mechanism on these sites. Interestingly the aliphatic carbons amino acids that are not directly linked to amino or hydroxyl groups also show sizable scalar ODNP enhancement (Fig. 2a–d). It seems that, besides the amino and hydroxyl groups that are well-known for their H-bonding capacities, aliphatic C−H groups also participate in the intermolecular interactions with TEMPOL radical.

**Fig. 3:**
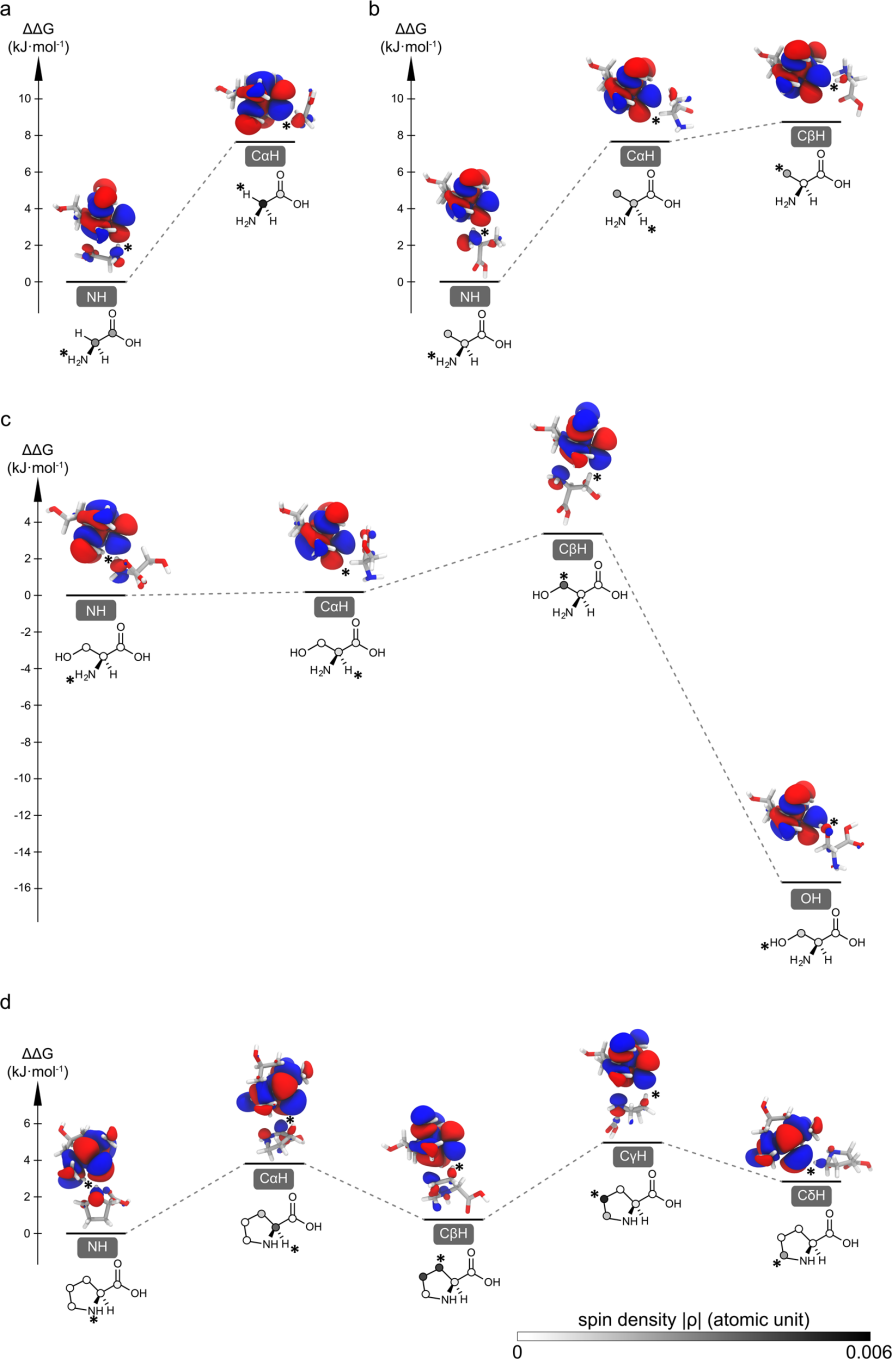
Chemical interactions between TEMPOL radical and amino acids mapped by DFT calculations. The structural and energy landscapes of H-bonded glycine (**a**), alanine (**b**), serine (**c**), proline (**d**)-TEMPOL complexes are presented. The relative Gibbs free energies (ΔΔ*G*) of the complexes involving different binding sites are referenced to the amino-binding state. The SOMO orbitals and the corresponding spin densities are presented along with the energy. The TEMPOL-substrate interaction sites are indicated with * symbol. The magnitude of spin densities on various carbons is presented in gray scale as shown in (**d**).

To further resolve the molecular mechanism of the observed site-specific ODNP enhancement in these amino acids, we have performed DFT calculations of these TEMPOL-amino acid complexes (Fig. 3 and Supplementary Table 5). For each amino acid, the amine group-mediated TEMPOL-amino acid complex has been taken as the reference for deriving the relative Gibbs free energy (ΔΔ*G*) of the other binding configurations. Our simulations support the binding of TEMPOL to the amino groups as shown by the lower Gibbs energy of the corresponding complexes (Fig. 3a–d). For serine, the complex organized by the sidechain hydroxyl group is significantly more stable than that via the amino group (ΔΔ*G* = −15.7 kJ/mol, Fig. 3c), which demonstrates the stronger H-bonding capacity of hydroxyl groups. This binding site preference agrees with the higher scalar ODNP enhancement of Cβ than that of Cα carbon in serine (Fig. 2a and Supplementary Table 3). The aliphatic C-H groups in all the explored amino acids also show certain radical-binding capacities (Fig. 3a–d, ΔΔG in the range of 0.8−8.7 kJ/mol) in line with the generally observed scalar ODNP enhancement on these carbons (Fig. 2a–d and Supplementary Table 3). For serine and proline, the aliphatic CH-organized TEMPOL-amino acid complexes only show slightly higher energies (ΔΔ*G* < 5 kJ/mol) than the NH-mediated complexes (Fig. 3c, d). Interestingly, our structure optimization of the TEMPOL-proline (CγHγ1) complexes has led consistently to a binding configuration to the neighboring CβHβ1 group, suggesting a competition or cooperativity between these two sites. Similarly, in the TEMPOL-proline(CβHβ2) complex the neighboring Cγ carbon also carries significant spin density (Fig. 3d and Supplementary Table 5). The relative Gibbs energies of the TEMPOL-glycine/alanine(C−H) complexes (ΔΔ*G* is close to 8 kJ/mol) are higher than those of the TEMPOL-serine/proline complexes. It has been reported that the methyl groups form weak H-bond with TEMPO-type radicals^55^. Our results agree with this previous finding and demonstrate that such methyl-radical interactions could lead to scalar ODNP enhancement. Compared to other amino acids, glycine shows significantly both higher $\overline{{\delta }_{{{{{{\rm{para}}}}}}}}$ (Supplementary Fig. 2f) and ODNP enhancement (Fig. 2c) on its Cα carbon (Fig. 2f and Supplementary Table 4). This is likely due to the lack of competing radical binding sites (sidechain carbons) in glycine. In summary, our simulations support that a wide range of chemical moieties, including the amine, hydroxyl, methyl, and aliphatic CH_2_ and CH groups, show binding capacity towards TEMPOL radical and therefore could all mediate the chemical engagement with the radicals for scalar ODNP.

### ODNP ^13^C NMR of biologically relevant heterocyclic compound indole

**Fig. 4:**
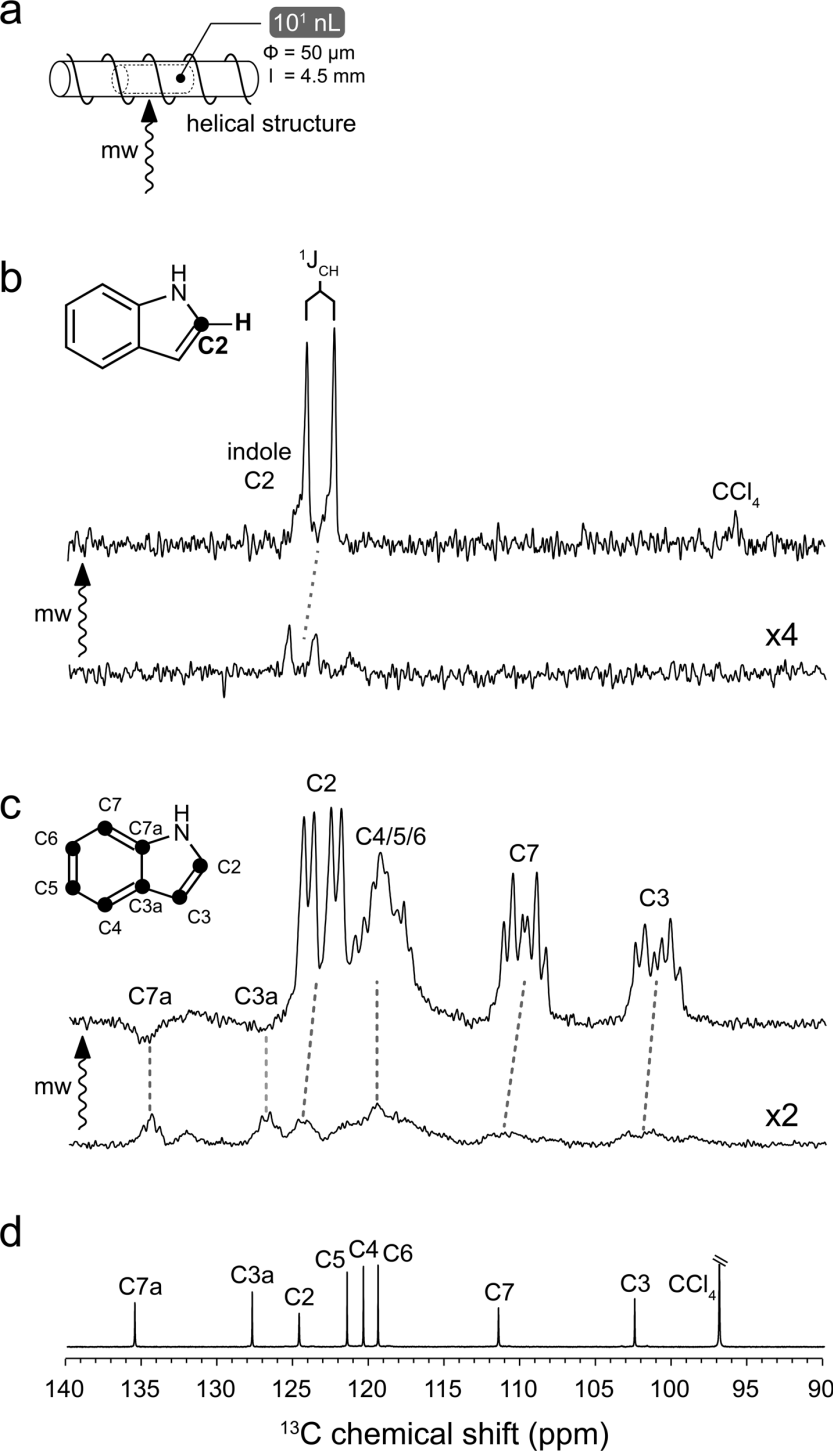
Room-temperature liquid-state ODNP NMR of indole in CCl_4_ at 9.4 T. TEMPO (100 mM) has been used as the hyperpolarizing agent. CCl_4_ has been chosen as the solvent. **a** Scheme of helical resonator^56^. The solution samples (10^1^ nL) have been loaded into a capillary of 50 μm diameter (Φ). The sample length (*l*) is about 4.5 mm. The microwave beam (mw arrow) enters the cavity from side. **b** Spectra of ^13^C2 indole obtained with (up) and without (down) microwave (mw arrow) are presented. The resolved J-coupling is indicated. **c** Spectra of U–^13^C indole obtained with (up) and without (down) microwave are presented. The molecular structures of ^13^C2 indole and U–^13^C indole are shown in the insets of panels (**b**) and (**c**) respectively. The ^13^C labeled positions are indicated by black circles. The spectra in (**b**) and (**c**) are scaled with the number of scans for visualizing directly the enhancement factor. The microwave (mw)-off spectra in (**b**) and (**c**) are further scaled up by four-fold and two-fold respectively for the better visualization. **d** The conventional ^1^H-decoupled liquid-state ^13^C NMR spectrum of indole without the ^13^C enrichment.

Biological molecules often bear heterocyclic moieties, such as imidazole in histidine, indole ring in tryptophan, and nucleobases in nucleic acids. Encouraged by the successful selection of imidazole as a scalar ODNP candidate from the previous paramagnetic NMR literature^53^, we decided to expand this endeavor to other biologically relevant heterocyclic compounds. In the original paramagnetic NMR work, from which imidazole has been sourced, a full list of nitrogenous heterocyclic compounds have been shown to interact with TEMPO radical via intermolecular H-bonds^53^. In particular indole (Fig. 4b, c), the sidechain moiety of tryptophan, interacts with TEMPO and shows rather large paramagnetic ^13^C NMR shifts (Supplementary Table 4)^53^. We, therefore, selected this compound for ODNP ^13^C NMR experiments. Due to the poor solubility of indole in water, we switched to the apolar CCl_4_ solvent. Previously we have developed another liquid-state ODNP NMR resonator at 9.4 T featuring a cylindrical microwave structure (Fig. 4a)^56^ that is highly suitable for the volatile CCl_4_ solvent^25^. We have therefore selected this setting for the ODNP ^13^C NMR experiments on indole. TEMPO at 100 mM has been used as the DNP agent in this experiment.

As shown in Fig. 4c, in the uniformly–^13^C ([U–^13^C]) labeled indole significant positive ODNP enhancements have been observed on all except the two bridge carbons (C3a and C7a). The positive enhancements point to a dominating scalar ODNP mechanism on the peripheral carbons, while the negative ^13^C enhancements indicate the prevailing dipolar ODNP mechanism on the bridge carbons. The ^13^C–^13^C homonuclear and ^1^H–^13^C heteronuclear J-coupling network in [U–^13^C] indole leads to rather complex spectroscopic patterns (Fig. 4c). Nevertheless, with the help of conventional ^1^H-decoupled liquid-state ^13^C NMR spectrum (Fig. 4d), an unambiguous assignment of most of the signals on the ODNP ^13^C NMR spectrum of [U–^13^C] indole can be achieved. To further resolve the spectroscopic complexity caused by the ^13^C–^13^C homonuclear J-coupling, we have turned to another labeling scheme with a single ^13^C site (^13^C2 indole, Fig. 4b). The ODNP-enhanced ^13^C NMR spectrum of ^13^C2-indole shows one ^13^C doublet (Fig. 4b). The resolution of the spectrum (33 Hz without ^1^H decoupling) permits to resolve the splitting of this ^13^C doublet and to quantify the corresponding H2-C2 ^1^J_CH_-coupling (181 Hz) (Fig. 4c). In addition, we have even detected the ODNP-enhanced NMR signal of CCl_4_ carbon (Fig. 4b) at its natural isotope abundance (ca. 0.15 M ^13^C spins) in the same sample.

The spectral resolution also allowed us to derive the site-specific ODNP enhancement factor for different carbons in the indole ring (Supplementary Table 4). Qualitatively the indole carbons exhibiting large $\overline{{\delta }_{{{{{{\rm{para}}}}}}}}$ values also show more evident ODNP enhancements except the two bridge carbons (Supplementary Fig. 3c and Supplementary Table 4). We have further expanded this analysis to other compounds (Supplementary Fig. 3a) previously studied by ODNP ^13^C NMR spectroscopy in organic solvents at the same magnetic field (9.4 T)^25^. Due to the recent in-house modification of the helical microwave structure (different plungers), we could only reach, though reproducibly, a moderately lower ODNP enhancement on the TEMPONE/^13^CCl_4_ sample compared to the previous work (Supplementary Fig. 1c)^25^. The ODNP enhancement of this new experimental result was used for rescaling the previous results in order to match the current instrumentation condition. As shown in Supplementary Fig. 3b and Supplementary Table 4, a correlation of ODNP enhancement over $\overline{{\delta }_{{{{{{\rm{para}}}}}}}}$ remains visible in this expanded molecular scheme. However, the data points seem more scattered than those shown in Fig. 2f. This could be due to the distinct nature (e.g., the halogen-bond and H-bond) of the radical-substrate interactions presented by this more diverse scheme of organic compounds. In addition, both the magnitude and the dynamics of hyperfine interactions determine the ODNP enhancement, while the paramagnetic NMR shift only indicates the average scale of hyperfine interactions. Since the dynamics of the H-bonded radical-substrate complexes had not been well explored, we decided to further explore such dynamics in the TEMPO-indole complex by advanced computational chemistry approaches.

### Molecular dynamics permits scalar ODNP in the indole-TEMPO complex

Since the binding chemistry between indole and TEMPO is well-defined, we selected this complex as our model system for a detailed investigation on the molecular dynamics relevant to scalar ODNP. The sub-ps dynamics of the TEMPO-indole complex can neither be accessed directly by EPR/paramagnetic NMR spectroscopy nor be depicted by conventional single-point QM calculations. In previous studies, the “pulse model” has been used to describe the microscopic mechanism of scalar ODNP^25,55^. In such a model the fluctuations of hyperfine interactions that drive ODNP are associated primarily with the transient formation-disassociation of radical-substrate complexes, which can be approximated as “pulses”^36,37^. We have estimated that the lifetime of the TEMPO-indole complex is longer than 100 ps (Supplementary Note 2), a time scale mismatching with high-field ODNP. To resolve the molecular mechanism of the Fermi contact fluctuations in the long-living TEMPO-indole complex, we have carried out an extensive in silico simulation.

**Fig. 5:**
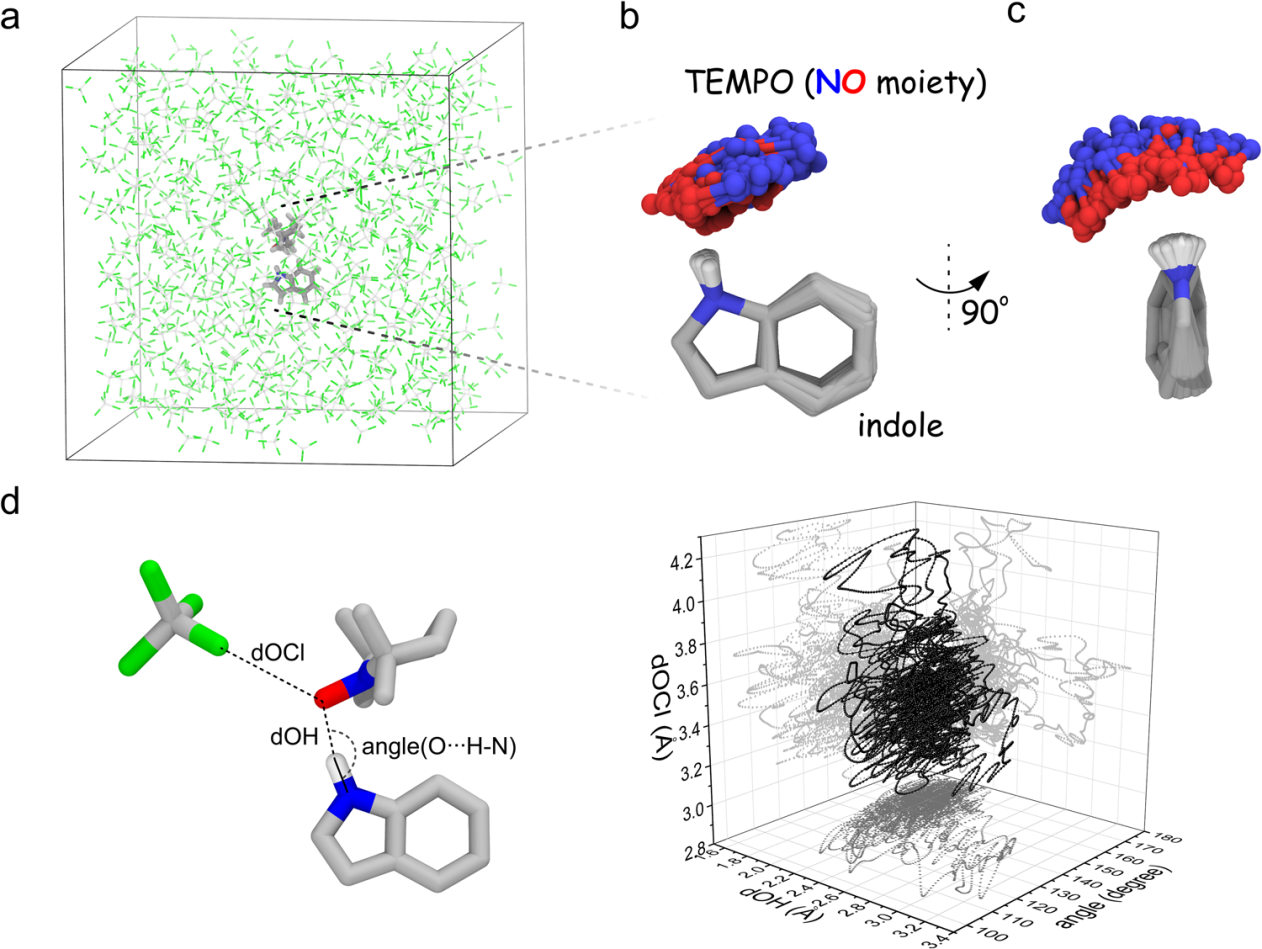
QM/MM MD simulation captures the structure dynamics of the H-bonded TEMPO-indole complex. **a** The TEMPO-indole complex (QM) is immerse in CCl_4_ solvent (MM) for the simulation. **b**, **c** Both the intermolecular H-bond and the indole ring undergo the structural fluctuations. **d** Both the H-bond (length and angle) and the radical-solvent distance undergo the dynamic fluctuations. Source data are provided as a Source Data file.

Since the Fermi contact is determined by the electronic structure of the complex, QM/MM MD simulation has been selected rather than the conventional MD approach based on classical mechanics. This ab initio approach also covers the contribution of the unpaired electron in the radical-substrate chemical interaction that cannot be depicted faithfully by empirical force fields. The TEMPO-indole complex (45 atoms) was included in the QM region set at the M06-2X/6-311G** level and the explicit solvent CCl_4_ bath (498 molecules, 2490 atoms) were treated using classical mechanics (Fig. 5a). We have chosen M06-2X functional for the QM simulation of H-bonded TEMPO-indole complex due to its excellent radical/nonradical trade-off, namely the balanced accuracy on both the open and closed-shell molecules^57^. For resolving the hyperfine fluctuations at the sub-ps time scale, we have simulated the QM/MM trajectory of 10 ps (1 × 10^4^ h CPU time). This extensive computational effort has yielded new structural, chemical, and dynamic insights into the molecular mechanism of high-field scalar ODNP.

First, our simulation reveals both the intermolecular and the intramolecular structural dynamics in the indole-TEMPO complex. The intermolecular H-bond in this complex fluctuates drastically and samples a large space of local geometry (Fig. 5b-d). In addition, the benzene and the pyrrole ring of the indole molecule also undergo fast intramolecular structural fluctuations (Fig. 5b, c). It has been reported that TEMPO-type radicals also interact chemically with CCl_4_ via halogen-mediated interactions^20,54^. In our simulation the average TEMPO oxygen–CCl_4_ chlorine distance (3.42 Å) remains significantly longer than that in TEMPONE–CCl_4_ complex (2.96 Å), suggesting a negligible role of nearby CCl_4_ molecules in competing for direct chemical interactions with the TEMPO radical within the time window of our simulation. The fluctuations of the surrounding CCl_4_ molecules may reshape the solvent environment for accommodating the dynamic TEMPO-indole complex.

**Fig. 6:**
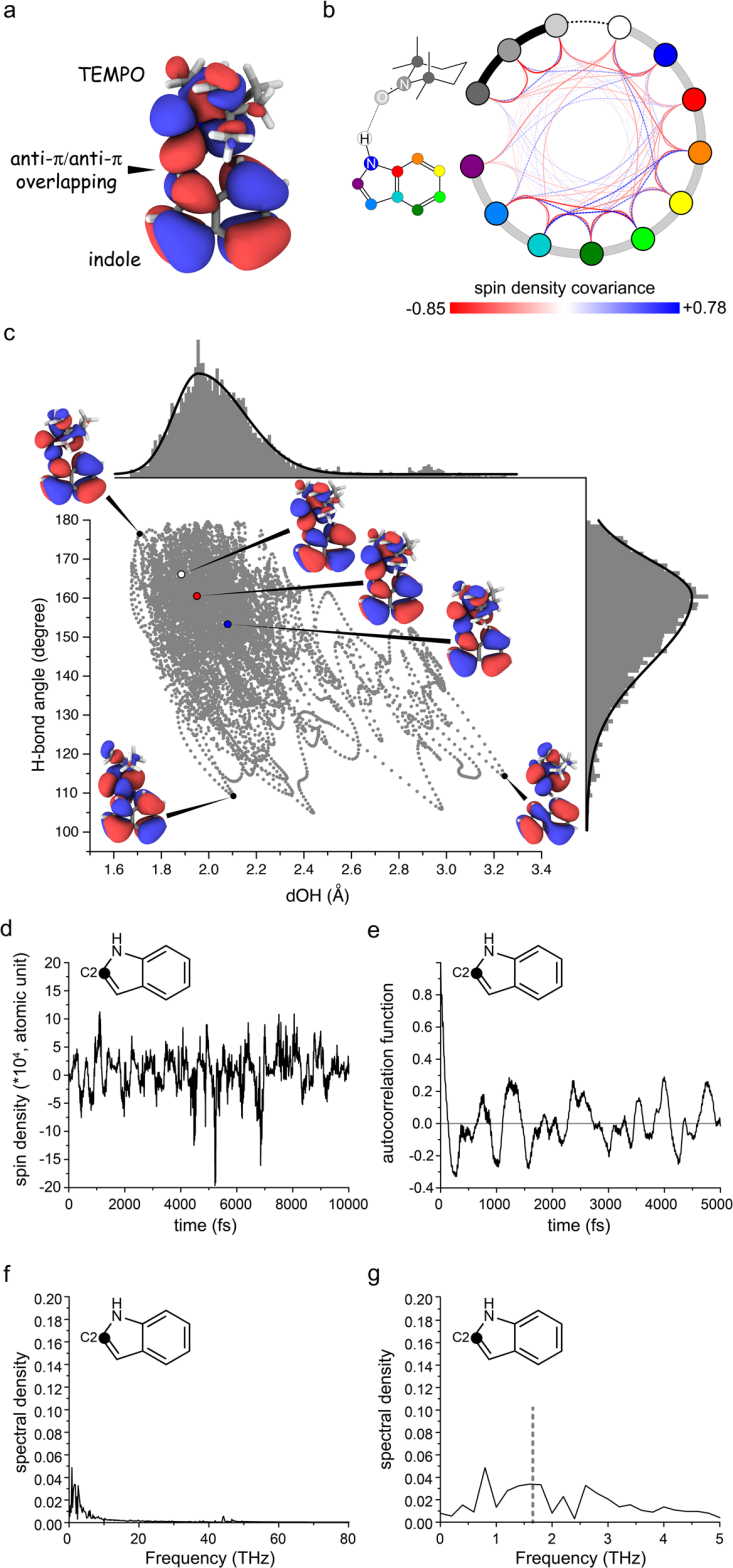
The dynamic electronic structures of the TEMPO-indole complex. **a** The SOMO of a representative conformation of the complex. **b** The covariance of spin density fluctuations at the various positions in the complex. The atoms are represented as the colored circles. The red and blue links indicate the negative and positive covariance respectively. **c** The SOMO of several states sampling distinct H-bond structures (length and angle) are shown. **d** The spin density trajectory of indole C2. **e** The autocorrelation function of the spin density dynamics at indole C2 position. **f**, **g** The unnormalized spectral density function of the spin density dynamics at indole C2 position. The dashed line in (**g**) indicates e-^13^C zero-quantum frequency at 9.4 T. Source data are provided as a Source Data file.

Second, the structural fluctuations within the TEMPO-indole complex are coupled with the spin density dynamics on indole carbons. As shown on a representative conformation of the TEMPO-indole complex (Fig. 6a), the singly occupied molecular orbital (SOMO) of the TEMPO radical overlaps with its indole counterpart in the H-bonded complex. The front lobe of the TEMPO SOMO located at the NO moiety overlaps with the indole SOMO in a head-to-side anti-π/anti-π configuration (Fig. 6a). In this configuration, the orbital overlap is sensitive to the intermolecular H-bond geometry. In addition, the indole SOMO orbital is also tuned by the indole ring structure (Fig. 6a). Through the intermolecular SOMO overlap, the electron spin density delocalizes from the TEMPO radical to the indole anti-π orbital (Fig. 6a). These spin densities further propagate to the indole ^13^C nuclear positions via the local anti-π/σ ortibal overlap. The structural fluctuations of both the TEMPO-indole H-bond and the indole ring itself contribute to the spin density dynamics on the indole carbons (Fig. 6c). Notably, the two bridge carbons in the indole molecule are located at the node position of its SOMO (Fig. 6a, c). Indeed they exhibit negligible ODNP ^13^C enhancements compared to the other carbons.

Third, the spin density dynamics within the TEMPO-indole complex present ODNP-relevant sub-ps features. The covariance between the spin density dynamics of various nuclei in the TEMPO-indole complex shows only a weekly correlated pattern (Fig. 6b). The individuality of the spin density dynamics on different indole carbons reflects the structural and chemical complexity of the Fermi contact in this complex as described above. To extract the time scale features of such complex spin density dynamics, we have performed a comprehensive analysis of the spin density trajectories (Supplementary Fig. 5) derived from our QM/MM MD simulation. Some detailed introductions on the theoretical and mathematical background of our data analysis can be found in Supplementary Information (Supplementary Note 4 and Supplementary Fig. 4) along with the full data presentation (Supplementary Figs. 5–11). The representative results of the spin density dynamics at the indole C2 position are shown in Fig. 6d–g.

The spin density autocorrelation functions (ACFs) of all indole carbons exhibit an initial decay followed by the sub-ps waves (Fig. 6e and Supplementary Fig. 6). The correlation times presented by the initial ACF decay are below 0.1 ps as extracted by the inverse Laplace transformation (Supplementary Fig. 8a–j). For most of the indole carbons, a second slower decaying component in the 0.5−1.0 ps range is also detectable (Supplementary Fig. 8b–j). The fast ACF decay at the similar time scale (0.1 ps) can be found on the intermolecular H-bond dynamics (Supplementary Fig. 8q, r) but not on the TEMPO-CCl_4_ (solute-solvent) fluctuation or on the TEMPO methyl rotation (Supplementary Figs. 5m–p, s, S7m–p, s). This further supports that the spin density dynamics in the TEMPO-indole complex is associated primarily with the intermolecular H-bond dynamics. The complex features of spin-density ACFs (Fig. 6e and Supplementary Fig. 6) fit neither the “pulse” models^36,37^ nor the commonly used memory-free Ornstein−Uhlenbeck process^58^. The spin density dynamics here can be better described by a more generalized autoregressive (AR) data model widely used in the time series analysis (Supplementary Note 4)^59^. The order of the AR model for describing the spin density dynamics in the TEMPO-indole complex is at least 6 (Supplementary Fig. 11q–t). This suggests a short “memory” of 6 fs (6 steps with 1 fs/step interval in our simulation) of the non-Markovian spin density dynamics, which aligns nicely with memory time at the 10 fs time scale as detected by the spin density memory functions. The detailed introduction and analysis of the memory function can be found in Supplementary Note 4 and Supplementary Figs. 9, 10 respectively.

Despite the highly complex nature of the spin density dynamics in the TEMPO-indole complex, we could derive the spectral density functions by Fourier transforming the spin density ACFs (Fig. 6f, g and Supplementary Figs. 4,  7). The spectral density at the e-^13^C ZQ frequency (0.263 * 2π = 1.65 THz) infers the ODNP performance. Indeed the e-^13^C ZQ relaxation rates estimated from the e-^13^C ZQ spectral density are in the order of magnitude of 10^−1^ to 10^1^ s^−1^ (Supplementary Note 5 and Supplementary Table 6), which agrees qualitatively with the order of magnitude of the observed DNP ^13^C enhancements.

## Discussion

In this work, we have discovered a full scheme of new molecular targets for scalar ODNP ^13^C NMR spectroscopy ranging from structurally and chemically diverse small biological molecules (carbohydrates and amino acids) to biologically relevant heterocyclic compounds (imidazole and indole). In particular, we have achieved sizable ODNP ^13^C enhancement on small biological molecules in water at room temperature and at high field (9.4 T). The paramagnetic NMR shift, a parameter that is rather easily accessible, has served us as an indicator for our initial search for such target molecules. Our data also identify a rather broad range of scalar ODNP-friendly chemical groups that interact with TEMPO-type radicals. The methyl groups, which are widely used NMR probes especially for large biomolecular complexes, form weak H-bonds with the TEMPO-type radicals and are eligible for room-temperature scalar ODNP. More generally, the H-bonding capacity of CH, OH, and NH groups with TEMPO-type radicals^55^, suggests that a broad range of organic molecules could be explored by scalar ODNP ^13^C NMR spectroscopy. We expect that paramagnetic NMR will continue to serve as a tool for facilitating the future ODNP target discovery. The spectral resolution achieved in our room-temperature ODNP ^13^C NMR experiments, even in the presence of high concentration (100 mM) of paramagnetic species, is already sufficient for resolving some ^1^J couplings. This opens the door towards more challenging ODNP 2D NMR experiments on such samples.

In previous ODNP studies^20,25,37^, the radical-substrate chemical encounter-disassociation events were considered as the main driving-force of scalar ODNP. In the halogen-bonded radical-substrate systems such events indeed occur frequently at the sub-ps time scale suitable for high-field scalar ODNP (Supplementary Note 3). In contrast, the lifetime of the indole-TEMPO H-bond complex is drastically longer than those halogen-bond complexes (Supplementary Note 2). The long lifetime in the 100 ps regime has also been proposed on some other H-bonded radical-substrate complexes^55^. This trend is in line with the generally stronger H-bond interaction than the halogen-bond in radical-substrate complexes as indicated by the binding constants (Supplementary Notes 2 and 3, 10^−1^ to 10^0^ M^−1^ for the H-bonded radical-substrate complexes and 10^−2^ M^−1^ for the halogen-bonded complexes). At low magnetic fields (e.g., 0.33 T, 10 GHz electron Larmor frequency), radical-substrate interaction-disassociation processes at 10^2^ ps time scale are fast enough for a good ODNP efficiency^40,55^. At high magnetic fields (e.g., 9.4 T in this study, 263 GHz electron Larmor frequency), molecular events at 10^2^ ps time scale is two orders of magnitude slower than those required for promoting efficient e-^13^C cross-relaxation. Rather the dynamic fluctuation within the long-living radical-substrate complex serves as the main driving force of scalar ODNP at such high magnetic field. Our simulation reveals that the intermolecular H-bond dynamics together with the intramolecular structural fluctuations are responsible for the high-field scalar ODNP in the long-living H-bonded TEMPO-indole complex. These molecular dynamics impact directly the intermolecular and intramolecular SOMO and are coupled with the spin density dynamics of the indole carbons. Such distinct molecular mechanisms in the H-bonded and halogen-bonded radical-substrate complexes could complicate the tentative correlation between ODNP enhancement and $\overline{{\delta }_{{{{{{\rm{para}}}}}}}}$. A more detailed discussion on additional factors that may also cause such deviations can be found in Supplementary Discussion.

Our QM/MM MD simulation also unfolds the sub-ps features of the spin density dynamics in a H-bonded radical-substrate complex. In the framework of an AR model, the sub-ps sinusoidal-like waves presented in the spin density ACFs are determined by the much shorter “memory” of the dynamic process. The origin of this memory, namely the intra-complex quantum mechanical interactions and other potential latent degrees of freedoms, will be explored in our future work. Molecular dynamics at the sub-ps time scale has been found in many H-bonded molecules by terahertz (THz) spectroscopy^60–66^. Our data analysis framework based on QM/MM MD trajectories offers a new approach for exploring the ODNP potential of such fast THz modes in the H-bonded molecular systems.

In summary, we show that the carbons in structurally and chemically diverse small biological and biologically-relevant molecules, ranging from heterocyclic compounds, carbohydrates to amino acids, can be hyperpolarized via scalar ODNP at room temperature. In particular, sizable DNP ^13^C enhancements, along with sufficient spectral resolution, can be obtained on small biological molecules in water directly at room-temperature and at high-field (9.4 T). All these observed scalar ODNP enhancements are based on intermolecular H-bonds in the radical-substrate complexes, suggesting a broad applicability of liquid-state ODNP ^13^C NMR spectroscopy. Paramagnetic NMR spectroscopy, a technique strongly related to DNP, can facilitate the ODNP target search. In addition, QM/MM MD simulations offer mechanistic insights into the fast sub-ps dynamics in such H-bonded radical-substrate complexes. We expect that our work will promote the exploration of other families of molecules for room-temperature liquid-state ODNP NMR applications and will encourage further developments in high-field DNP instrumentation, QM/MM MD simulations, and the use of other complementary approaches for detecting and describing ultrafast H-bond dynamics in liquids.

## Methods

### Materials and sample preparation

U-^13^C indole, U-^13^C glucose, and ^13^CCl_4_ were purchased from Cambridge Isotope Laboratory (Tewksbury, U.S.). ^13^C-imidazole was obtained from Toronto Research Chemicals TRC (I350204, North York, Canada). ^13^C-labeled amino acids and D_2_O ( > 99%) were obtained from CortecNet (Les Ulis, France). TEMPO, TEMPOL and TEMPONE were obtained from Aldrich-Sigma and were used without further purification. The solvent CCl_4_ was purchased form ABCR (Karlsruhe, Germany). Radical and ^13^C-enriched compounds were dissolved together in a specific solvent and all samples were used as soon as prepared or immediately stored at −80 °C before the further use. We have found that imidazole could not reach a good concentration in CCl_4_. Therefore chloroform was used instead of CCl_4_ for this compound. The details about all ODNP NMR samples are listed in Supplementary Table 1.

For paramagnetic NMR titration experiments, all samples were prepared using compounds of natural ^13^C abundance at the concentrations in Supplementary Table 1 or reported previously^25^. The concentrations of radicals were varied between 0 and 200 mM. The samples were loaded to brown-colored 5 mm NMR tubes for the solution NMR measurements. The frequency locking on the deuterium-free sample (e.g., CCl_4_) was achieved by placing an external DMSO-d_6_ sample sealed in 1.0 mm (outer diameter) capillary into the solution NMR tubes.

### ODNP experiments and data analysis

All liquid-state DNP experiments were performed on a homebuilt DNP NMR spectrometer operating at 9.4 T (^1^H Larmor frequency 400 MHz, electron Larmor frequency 263 GHz)^56^. Microwave (MW) irradiation was generated in a customized 4.7 T gyrotron (Gycom, Russia) operating at the second harmonic mode and was directed to DNP NMR probe via corrugated waveguides. The maximal microwave power from the MW bridge was 5.5 W. A commercial Bruker Avance 9.4 T spectrometer was used for the NMR experiments.

DNP experiments on aqueous samples were carried out on a stripline Fabry–Pérot (FP) DNP NMR probehead modified from the previous design^39^. The Fabry–Pérot cavity offered high microwave efficiency on thin flat samples while restricting the sample heating. The stripeline served as the RF component for the NMR experiment. The sample solution was first loaded onto the stripline using a 1 μL microsyringe (Hamilton) and then sealed with a flat Teflon ring (2 mm inner diameter, 15 μm thickness) under a quartz plate (Stellar Industries Corp), which corresponds to an effective sample volume of 50 nL. The Teflon ring was pre-lubricated with proton-free grease (Crytox, DuPont) to prevent the leakage of the sample solution. The grease was not compatible with an organic solvent. Therefore the current setup is mostly suitable for aqueous samples. After being sealed, the samples were inspected carefully under microscope for the bubbles. Thanks to the small sample dimension (thickness) and high-heat conductance of metal stripeline, this FP probehead shows excellent heat management capacity, which permits the stable DNP NMR measurements on aqueous samples. The NMR spectra with thermal polarization (“Boltzmann” condition) were acquired without microwave irradiation. The DNP-enhanced NMR spectra were acquired under maximal MW power (5.5 W) obtainable from our gyrotron. The DNP working condition was optimized using a two-step procedure similar as that used on HC probehead (Supplementary Fig. 1a), with the exception that the distance between the stripeline and spherical mirror was adjusted for tuning the FP cavity. We noticed that the control of sample dimension could be helpful for maintaining the quality factor of the current FP cavity. The saturation factor under our experimental condition was estimated to be about 0.6 based on the observed DNP enhancement of water ^1^H (about −10 at 320 K). The RF channel can be switched to either ^1^H or ^13^C Larmor frequency. The ^13^C and ^1^H 1D NMR spectra were acquired and processed as described above. All ^1^H and ^13^C chemical shifts were referenced indirectly to TMS.

DNP experiments on samples with organic solvents were performed on a home-built helix-cylindrical (HC) ODNP NMR probehead, which features a cylindrical MW cavity and a helical radiofrequency (RF) coil^56^. Liquid samples were loaded in a quartz capillary (Polymicro, 50 or 100 μm inner diameter, 150 μm outer diameter) via the capillary effect and were sealed with wax on both ends. An air gap was left between the sample solution and the wax in order to prevent the direct contact between the organic solvent and the wax sealing. Before each measurement, the capillaries were carefully inspected for ensuring the absence of bubbles inside the sample volume. The sample heating could generate bubbles inside the sample or even cause the sample leakage. Such sample deteriorations were indicated by the loss of NMR signals. We observed that polar solvents in particular water with a high dielectric constant can be heated rapidly by MW in HC probehead. Small sample size (capillary diameter) would alleviate partially this heating issue. However, this would severely compromise the NMR sensitivity. Therefore on polar solvent samples, only qualitative results can be obtained on this probehead. The sample capillaries were placed horizontally along the axis of helix coil. The MW resonator was tuned to TE_013_ mode by adjusting the distance between two plungers made of Kel-F with silver-coated caps. The length of the MW cavity was about 4.5 mm, which corresponds to the active sample volume of 35 nL or 9 nL for 100 μm or 50 μm capillaries respectively. The NMR spectra with thermal polarization (“Boltzmann” condition) were acquired without microwave irradiation. The DNP-enhanced NMR spectra were acquired under 1.4 W microwave power. The best DNP working condition for each sample was located in two steps. First, the microwave frequency was first tuned preliminarily by scanning the gyrotron cavity temperature for the best DNP-enhancement on a standard TEPONE-^13^CCl_4_ sample. (Supplementary Fig. 1a) In the second step, the target sample was loaded and the cylinder microwave cavity was fine-tuned for each specific sample by adjusting the distance between plungers following the ^1^H signal of the sample. The RF channel was switched manually between ^1^H and ^13^C Larmor frequencies by reconfiguring the RF circuit inside probehead. For the ^13^C direct excitation NMR experiments, a “hard” ^13^C pulse π/2 pulse of 22.7 kHz RF strength was used for excitation. The carrier frequency for ^13^C RF pulse was set to 104 ppm. No ^1^H decoupling was applied during the acquisition. The recycle delay was set to 1 s that is long enough for the full ^13^C magnetization recovery. The ^13^C spectral window was set to 140 ppm (14.1 kHz) and the FID was digitalized with 8192 points. The FID was processed with 16k points with Gaussian window function (lb = −5 Hz, gb = 0.04). For the ^1^H NMR experiments, a ^1^H pulse π/2 pulse of 25.0 kHz RF strength was applied. The carrier frequency for ^1^H RF pulse was set to 6 ppm. The recycle delay was set to 1 s. The spectral window was set to 40 ppm (16.0 kHz) and the FID was digitalized with 4096 points. The FID was processed with 16k points without applying window functions. All ^1^H and ^13^C chemical shifts were referenced indirectly to TMS.

The ODNP enhancement factor was calculated with the following equation:

$${\varepsilon }_{{{{{{\rm{OE}}}}}}}=\frac{{I}_{{{{{{\rm{DNP}}}}}}}}{{I}_{{{{{{\rm{Boltzmann}}}}}}}}\cdot \frac{{n}_{{{{{{\rm{Boltzmann}}}}}}}}{{n}_{{{{{{\rm{DNP}}}}}}}}-1$$

where *I* is the integral of the signal and n is the number of scans.

The error of DNP enhancement was calculated by the signal-to-noise ratio of the NMR spectra using Gaussian error propagation law:

$$\varDelta {\varepsilon }_{{{{{{\rm{OE}}}}}}}=\sqrt{{\left(\frac{\partial {\varepsilon }_{{{{{{\rm{OE}}}}}}}}{\partial {I}_{{{{{{\rm{DNP}}}}}}}}\right)}^{2}{\varDelta {I}_{{{{{{\rm{DNP}}}}}}}}^{2}+{\left(\frac{\partial {\varepsilon }_{{{{{{\rm{OE}}}}}}}}{\partial {I}_{{{{{{\rm{Boltz}}}}}}}}\right)}^{2}{\varDelta {I}_{{{{{{\rm{Boltz}}}}}}}}^{2}}$$

where Δ*I* is the reciprocal of signal-to-noise ratio.

We found that the achievable ODNP enhancement on the same ^13^CCl_4_/TEMPONE sample had dropped significantly from the previous record obtained on the sample HC probehead^25^. Since the radical concentration was identical in all samples, we derived a scaling factor from the DNP enhancements of ^13^CCl_4_/TEMPONE sample under different HC probehead conditions and applied this scaling factor on previously reported results for a fairer comparison.

### Determination of molar-free paramagnetic chemical shifts ($\overline{{\delta }_{{{{{{\rm{para}}}}}}}}$)

Solution 1D ^13^C NMR experiments were performed on a Bruker 400 MHz spectrometer (Institute for Organic Chemistry and Chemical Biology, Goethe University of Frankfurt am Main). All ^1^H and ^13^C NMR experiments were performed using the standard setups at room temperature. ^13^C FIDs were acquired with ^1^H decoupling. All these solution experiments on the commercial instrument including the data processing were automated. The resonance assignment was performed manually and the molar-free paramagnetic shifts were obtained from the slope of the linear fitting of observed paramagnetic shift versus the radical concentration. The error of $\overline{{\delta }_{{{{{{\rm{para}}}}}}}}$ is defined as the data fitting error of this linear regression. For the cases where several carbon signals are overlapped on OD NP NMR spectra, we calculated the standard deviations of $\overline{{\delta }_{{{{{{\rm{para}}}}}}}}$ of these carbons and present this deviation as the error.

### Single point DFT, QM/MM MD simulation, and trajectory data extraction

The structures of TEMPOL-amino acid complexes were optimized in water phase at the M06-2X/6–311G** level using Gaussian16^67^. All the molecular orbitals were described by cubegen module of Gaussian program. The solvation effect was introduced via polarizable continuum model (PCM). We performed an exhaustive configurational search for the TEMPOL-amino acid complexes at each binding site, and kept the lowest-energy configuration. The vibrational frequencies were calculated to confirm the local minima with all positive frequencies. The Gibbs free energies (including the solvation energy) of the TEMPOL-amino acid complexes are given in Supplementary Table 5.

The quantum mechanics/molecular mechanics molecular dynamics (QM/MM MD) simulation of indole-TEMPO complex was performed with the explicit CCl_4_ solvent environment. The initial TEMPO-indole complex was constructed starting from the TEMPO-CHCl_3_ complex^20^ by replacing CHCl_3_ with indole. Subsequently, the TEMPO-indole complex was optimized in a carbon tetrachloride solvent environment at the M06-2X/6–311 G** level^68^ using the Gaussian16 program^67,68^. Polarizable Continuum Model (PCM) was applied to mimic the solvent environment.

Next, the TEMPO-indole complex was placed at the center of a rectangular box containing 498 carbon tetrachloride molecules. Force field parameters for carbon tetrachloride and the TEMPO-indole complex were taken from the Generalized Amber force field (GAFF)^69^ with the HF/6–31G* RESP charges. Minimization using the Amber force field was first performed to relax the system with a weak constraint. Then the system was brought to room temperature (300 K) in 100 ps with a weak constraint. After that, 100 ps classical MD simulation of the weakly restrained TEMPO-indole complex was carried out to further relax the system with the periodic boundary condition at 300 K and 1 atm. The integration time step was set to 1.0 fs.

Finally, 10 ps QM/MM MD simulation was performed after the pre-equilibrium simulation.

Currently, this simulation is set as a long-run task on the computational platform and a glimpse into the 20 ps result can be found in Supplementary Figs. 5t and  6t. The TEMPO-indole complex was partitioned into the QM region and the rest of the system was treated by MM. The QM region was calculated by M06–2X/6–311G**^68^. The electronic coupling between the QM and MM regions was treated by including the MM charges in the QM Hamiltonian. A 15 Å cutoff was utilized to treat QM/MM electrostatic interactions. The integration time step for QM/MM MD simulation was also set to 1.0 fs. The atomic spin densities of the TEMPO-indole complex were obtained from the QM/MM calculations. The Amber18 program^70^ was utilized to perform the MD simulations, and the Sander module with an interface to the Gaussian16 program was employed to carry out QM/MM MD simulations. The 10 ps trajectory calculation took 1 × 10^4^ h CPU (Intel Xeon E5–2650 2.30 GHz) time on our cluster. The time evolutions of the spin density and molecular geometry (H-bond length, H-bond angle, TEMPO-CCl_4_ distance, methyl rotation angle) were extracted from the QM/MM MD trajectory using in-house scripts. The 3D profiles of SOMO of selected conformations/frames were generated using GaussView. The QM/MM simulation was conducted on 20-core Intel Xeon E5–2650 2.30 GHz processors at the Supercomputer Center of East China Normal University (ECNU).

### Data analysis of QM/MM MD trajectory

A general introduction of the data analysis procedure is shown in Supplementary Fig. 4. The normalized autocorrelation functions (ACF) *g*(*t*) were calculated from the QM/MM MD trajectory data following the definition below:

$$g(t)=\frac{ < \rho ({t}_{0})\rho ({t}_{0}+t) > }{ < \rho ({t}_{0})\rho ({t}_{0}) > }$$

where *ρ*(*t*) presents the specific target parameter (e.g., spin density, distance, angle) at time *t*. ACFs were computed from the corresponding trajectory data by a in-house written python script. In order to prevent the artificial “convergence” to zero caused by the finite length of trajectories, we only calculated ACF up to half of the trajectory time duration. High precision (100 decimals) was set for the ACFs in order to reduce the propagation of numerical errors from ACF to the later computed memory functions. In a test calculation, we also removed two methyl groups on TEMPO in QM/MM calculations and found that the spin density ACF shows similar pattern on indole in complex with this hypothetical radical.

All Fourier transforms were performed using Origin (OriginLab Corporation). The covariances of spin density variations between different atoms were calculated using StatPlus:mac (AnalystSoft Inc.) and were visualized using Circos–0.69–3^71^. The inverse Laplace transform of ACF was computed using CONTIN^72^ as a plug-in of Origin (https://www.originlab.com/fileExchange/details.aspx?fid=456).

The memory function *K*(*t*) was defined as below:

$$\frac{\partial }{\partial t}g(t)=-\int_{0}^{t}{K}_{g}(\tau )g(t-\tau ){{{{{\rm{d}}}}}}\tau$$

where *g*(*t*) is the ACF and *K*(*τ*) is its memory kernel. This definition is in the form of Volterra integral equation of the second type, in which the right side of the equation is also called Bromwich integral. The solution of this integral equation takes the form of Laplace and inverse Laplace transform:

$${K}_{g}(\tau )={ {\mathcal L} }^{-1}[{( {\mathcal L} [g(t)])}^{-1}-t]$$

where ${\mathcal L}$ and ${ {\mathcal L} }^{-1}$ presents the Laplace and inverse Laplace transform.

Initially, we attempted to compute the memory function by discrete Z-transform and inverse Z-transform. However, the computational burden became astronomical for a 10^4^ data sequence. Only the truncation of up to 20 terms was affordable for the initial points of the memory function on a normal PC. However, the error induced by such truncation escalated rapidly with increasing time (Supplementary Fig. 8p). Therefore the memory functions were eventually computed numerically following the protocol proposed by Berne and co-workers^73^ using a python script written in house. The Gregory formula was taken for approximating the integration in Day’s method for solving the linear Volterra integral equation^74^. To reduce the numerical error accumulated in the iteration, the decimal module was used for suppressing the rounding error of binary representation. We also calculated the memory function by reverting the Berne’s approach for deriving g(*t*) from K(*t*)^73^. With the decimal module, this approach yields the result similar to that obtained from the direct treatment of Volterra integral equation. Though the deviation between two numerical memory functions yielded by these two approaches indeed propagates with time/iteration, its absolute value remains negligible (Supplementary Fig. 9q). In this work, we still used the approach by solving the Volterra integral equation. It should be noticed that even with the best numerical treatment we can afford, the numerical error still propagates significantly on several sites as shown in Supplementary Fig. 9b, k, l, n, o.

The autoregressive AR-model-based time series analysis was performed using Mathematica (version 10.3.0.0). The function “TimeSeriesModelFit” was used and the model was set to “AR”. We have also tested more general models (Supplementary Fig. 11q–t). The best candidate was ARMA(6,1) (AR(6) with moving average MA(1)), the Akaike information criterion (AIC) of this model was −236421, which is just slightly better than AR(6) model (AIC −235660). Since the physical meaning of MA is difficult to be interpreted in our case and AR(6) model already captures the feature of the trajectory, we finally selected AR(6) model to represent the trajectory. We have also confirmed the stationarity of this model using the “WeakStationarity” function. Our analysis also shows that the AR models of orders p higher than 6 do not further improve the fitting quality and the six time-point correlation parameters along with the noise level are already converged at *p* = 6. Therefore *p* = 6 shows the minimal order of RA for representing our trajectory data. However, high order *p* > 6 still yields essentially non-zero time-point correlation parameters. Therefore *p* = 6 only defines the minimal order of time-point correlation in our data.

## Supplementary information

Supplementary Information

## Data Availability

The ODNP NMR data have been deposited in Figshare (10.6084/m9.figshare.14774433, 10.6084/m9.figshare.14774415). The results of DFT calculations have been deposited in GitHub (https://github.com/xiaohegroup/Simulations-of-Tempol). The full QM/MM trajectory is available from the corresponding authors upon reasonable requests. Source data are provided with this paper.

## Source data

Source Data

## Code availability

All input files for the DFT calculations and QM/MM simulations are available via GitHub (https://github.com/xiaohegroup/Simulations-of-Tempol, 10.5281/zenodo.5150908^75^). The codes for analyzing the QM/MM trajectory are available from the corresponding authors upon reasonable requests.

## References

[1] Ardenkjaer-Larsen JH (2015). Facing and overcoming sensitivity challenges in biomolecular NMR spectroscopy. Angew. Chem. Int. Ed..

[2] Ni QZ (2013). High frequency dynamic nuclear polarization. Acc. Chem. Res..

[3] Lelli M (2015). Solid-State dynamic nuclear polarization at 9.4 and 18.8 T from 100 K to room temperature. J. Am. Chem. Soc..

[4] Biller JR, Barnes R, Han S (2018). Perspective of Overhauser dynamic nuclear polarization for the study of soft materials. Curr. Opin. Colloid Interface Sci..

[5] Saliba EP, Sesti EL, Alaniva N, Barnes AB (2018). Pulsed electron decoupling and strategies for time domain dynamic nuclear polarization with magic angle spinning. J. Phys. Chem. Lett..

[6] Zhao L, Pinon AC, Emsley L, Rossini AJ (2018). DNP-enhanced solid-state NMR spectroscopy of active pharmaceutical ingredients. Magn. Reson. Chem..

[7] Denysenkov VP, Prisner TF (2019). Liquid-state Overhauser DNP at high magnetic fields. Emagres.

[8] Jannin S, Dumez JN, Giraudeau P, Kurzbach D (2019). Application and methodology of dissolution dynamic nuclear polarization in physical, chemical and biological contexts. J. Magn. Reson..

[9] Corzilius B (2020). High-field dynamic nuclear polarization. Annu. Rev. Phys. Chem..

[10] Prandolini MJ, Denysenkov VP, Gafurov M, Endeward B, Prisner TF (2009). High-field dynamic nuclear polarization in aqueous solutions. J. Am. Chem. Soc..

[11] Lesage A (2010). Surface enhanced NMR spectroscopy by dynamic nuclear polarization. J. Am. Chem. Soc..

[12] Lumata L, Merritt ME, Hashami Z, Ratnakar SJ, Kovacs Z (2012). Production and NMR characterization of hyperpolarized (107,109)Ag complexes. Angew. Chem. Int. Ed..

[13] Franck JM, Pavlova A, Scott JA, Han S (2013). Quantitative cw Overhauser effect dynamic nuclear polarization for the analysis of local water dynamics. Prog. Nucl. Magn. Reson. Spectrosc..

[14] Takahashi H, Hediger S, De Paepe G (2013). Matrix-free dynamic nuclear polarization enables solid-state NMR 13C-13C correlation spectroscopy of proteins at natural isotopic abundance. Chem. Commun..

[15] Can TV (2014). Overhauser effects in insulating solids. J. Chem. Phys..

[16] Jakdetchai O (2014). Dynamic nuclear polarization-enhanced NMR on aligned lipid lilayers at ambient temperature. J. Am. Chem. Soc..

[17] Harris T, Szekely O, Frydman L (2014). On the potential of hyperpolarized water in biomolecular NMR studies. J. Phys. Chem. B.

[18] Valentine KG (2014). Reverse micelles as a platform for dynamic nuclear polarization in solution NMR of proteins. J. Am. Chem. Soc..

[19] Wang X (2015). Optimization and prediction of the electron-nuclear dipolar and scalar interaction in H-1 and C-13 liquid state dynamic nuclear polarization. Chem. Sci..

[20] Liu GQ (2017). One-thousand-fold enhancement of high field liquid nuclear magnetic resonance signals at room temperature. Nat. Chem..

[21] Bielytskyi P (2018). 13C → 1H transfer of light-induced hyperpolarization allows for selective detection of protons in frozen photosynthetic reaction center. J. Magn. Reson..

[22] Dubroca T (2018). A quasi-optical and corrugated waveguide microwave transmission system for simultaneous dynamic nuclear polarization NMR on two separate 14.1 T spectrometers. J. Magn. Reson..

[23] Dubroca T, Wi S, van Tol J, Frydman L, Hill S (2019). Large volume liquid state scalar Overhauser dynamic nuclear polarization at high magnetic field. Phys. Chem. Chem. Phys..

[24] Narasimhan S (2019). DNP-supported solid-state NMR spectroscopy of proteins inside mammalian cells. Angew. Chem. Int. Ed..

[25] Orlando T (2019). Dynamic nuclear polarization of C-13 nuclei in the liquid state over a 10 Tesla field range. Angew. Chem. Int. Ed..

[26] Olsen GL (2020). Sensitivity-enhanced three-dimensional and carbon-detected two-dimensional NMR of proteins using hyperpolarized water. J. Biomol. NMR.

[27] Wang Y, Kim J, Hilty C (2020). Determination of protein-ligand binding modes using fast multi-dimensional NMR with hyperpolarization. Chem. Sci..

[28] Ardenkjaer-Larsen JH (2003). Increase in signal-to-noise ratio of > 10,000 times in liquid-state NMR. Proc. Natl Acad. Sci. USA.

[29] Dzien P (2016). Following metabolism in living microorganisms by hyperpolarized H-1 NMR. J. Am. Chem. Soc..

[30] Novakovic M (2020). A 300-fold enhancement of imino nucleic acid resonances by hyperpolarized water provides a new window for probing RNA refolding by 1D and 2D NMR. Proc. Natl Acad. Sci. USA.

[31] Otikovs M, Olsen GL, Kupce E, Frydman L (2019). Natural abundance, single-scan C-13-C-13-based structural elucidations by dissolution DNP NMR. J. Am. Chem. Soc..

[32] Szekely O, Olsen GL, Novakovic M, Rosenzweig R, Frydman L (2020). Assessing site-specific enhancements imparted by hyperpolarized water in folded and unfolded proteins by 2D HMQC NMR. J. Am. Chem. Soc..

[33] Krummenacker JG, Denysenkov VP, Prisner TF (2012). Liquid state DNP on metabolites at 260 GHz EPR/400 MHz NMR frequency. Appl. Magn. Reson..

[34] Neugebauer P (2013). Liquid state DNP of water at 9.2 T: an experimental access to saturation. Phys. Chem. Chem. Phys..

[35] Neugebauer P (2014). High-field liquid state NMR hyperpolarization: a combined DNP/NMRD approach. Phys. Chem. Chem. Phys..

[36] Noack F, Krüger GJ, Müller-Warmuth W, Van Steenwinkel R (1967). Stochastische Prozesse in Spinsystemen. Z. Naturforsch. A.

[37] Müller-Warmuth W, Van Steenwinkel R, Noack F (1968). Dynamic nuclear polarization experiments on 19F in solutions and their interpretation by the ‘pulse model’ of molecular collisions. Z. Naturforsch. A.

[38] Liebe HJ, Hufford GA, Manabe T (1991). A model for the complex permittivity of water at frequencies below 1 THz. Int. J. Infrared Millim. Waves.

[39] Denysenkov V, Prisner T (2012). Liquid state dynamic nuclear polarization probe with Fabry−Perot resonator at 9.2 T. J. Magn. Reson..

[40] Dorn HC, Gu J, Bethune DS, Johnson RD, Yannoni CS (1993). The nature of fullerene solution collisional dynamics—a C-13 DNP and NMR-study of the C60/C6D6/TEMPO system. Chem. Phys. Lett..

[41] Banci L, Camponeschi F, Ciofi-Baffoni S, Piccioli M (2018). The NMR contribution to protein−protein networking in Fe−S protein maturation. J. Biol. Inorg. Chem..

[42] Bertini I (2010). Ultrafast MAS solid-state NMR permits extensive C-13 and H-1 detection in paramagnetic metalloproteins. J. Am. Chem. Soc..

[43] Bertini, I., Luchinat, C. & Parigi, G. *Solution NMR of Paramagnetic Molecules: Applications to Metallobiomolecules and Models* (Elsevier Science Ltd., 2001).

[44] Pell AJ, Pintacuda G, Grey CP (2019). Paramagnetic NMR in solution and the solid state. Prog. Nucl. Magn. Reson. Spectrosc..

[45] Westler WM, Lin IJ, Perczel A, Weinhold F, Markley JL (2011). Hyperfine-shifted C-13 resonance assignments in an iron-sulfur protein with quantum chemical verification: Aliphatic C-H center dot center dot center dot S 3-center-4-electron interactions. J. Am. Chem. Soc..

[46] Morishima I, Endo K, Yonezawa T (1971). Nuclear magnetic resonance contact shifts induced by hydrogen bonding with organic radicals. I. Proton and carbon-13 contact shifts of protic molecules in the presence of the nitroxide radical. J. Am. Chem. Soc..

[47] Morishima I, Inubushi T, Endo K, Yonezawa T, Goto K (1972). Interaction between closed- and open-shell molecules. VII. Carbon-13 contact shift and molecular or obital studies on the charge-transfer interaction between halogenated molecules and nitroxide radical. J. Am. Chem. Soc..

[48] Morishima I, Inubushi T, Yonezawa T (1976). Stable free-radicals as NMR spin probe for studying intermolecular interactions .12. C-13 contact shifts and electron-spin delocalization induced by charge-transfer interaction between halogenated molecules and stable free-radicals. J. Am. Chem. Soc..

[49] Morishima I, Inubushi T, Yonezawa T, Kyogoku Y (1977). Proton magnetic-resonance studies of specific association of nucleic-acid constituent bases in a non-aqueous solvent—utility of DTBN radical to probe affinity of hydrogen-bonding involved in complementary base-pairs. J. Am. Chem. Soc..

[50] Morishima I, Okada K, Yonezawa T (1972). Nuclear magnetic resonance contact shifts, and electron spin distribution. Proton and carbon-13 contact-shift studies of azanaphthalenes. J. Am. Chem. Soc..

[51] Morishima I, Toyoda K, Yoshikawa K, Yonezawa T (1973). Interaction between closed-shell and open-shell molecules—nuclear nagnetic-resonance contact shift studies on Pi-hydrogen bonding involving stable hydrocarbon Pi radicals. J. Am. Chem. Soc..

[52] Morishima I, Yonezawa T, Goto K, Imanari M, Kawakami K (1972). Interactions between closed-shell and open-shell molecules—C-13 contact shift studies on interaction between aromatic-hydrocarbons and nitroxide radical. J. Am. Chem. Soc..

[53] Qiu ZW, Grant DM, Pugmire RJ (1984). Paramagnetic C-13 shifts induced by the free-radical Tempo .2. nitrogen-heterocycles. J. Am. Chem. Soc..

[54] Brintzinger HH, Marvich RH (1971). Metastable form of titanocene—formation from a hydride complex and reactions with hydrogen, nitrogen, and carbon monoxide. J. Am. Chem. Soc..

[55] Russ JL (2007). Nitroxide/substrate weak hydrogen bonding: attitude and dynamics of collisions in solution. J. Am. Chem. Soc..

[56] Denysenkov VP (2008). High-field DNP spectrometer for liquids. Appl. Magn. Reson..

[57] Janesko BG, Verma P, Scalmani G, Frisch MJ, Truhlar DG (2020). M11plus, a range-separated hybrid meta functional incorporating nonlocal rung-3.5 correlation, exhibits broad accuracy on diverse databases. J. Phys. Chem. Lett..

[58] Abragam, A. *The Principles of Nuclear Magnetism* (Clarendon Press, 1961).

[59] Cryer, J. D. & Chan, K. S. *Time Series Analysis: With Applications in R* 2nd edn (Springer-Verlag, 2008).

[60] Ebbinghaus S (2007). An extended dynamical hydration shell around proteins. Proc. Natl Acad. Sci. USA.

[61] He X, Sode O, Xantheas SS, Hirata S (2012). Second-order many-body perturbation study of ice Ih. J. Chem. Phys..

[62] Nibali VC, Havenith M (2014). New insights into the role of water in biological function: studying solvated biomolecules using terahertz absorption spectroscopy in conjunction with molecular dynamics simulations. J. Am. Chem. Soc..

[63] Sun J (2014). Understanding THz spectra of aqueous solutions: glycine in light and heavy water. J. Am. Chem. Soc..

[64] Xu Y, Havenith M (2015). Perspective: Watching low-frequency vibrations of water in biomolecular recognition by THz spectroscopy. J. Chem. Phys..

[65] Liu JF, He X, Zhang JZH, Qi LW (2018). Hydrogen-bond structure dynamics in bulk water: insights from ab initio simulations with coupled cluster theory. Chem. Sci..

[66] Grechko M (2018). Coupling between intra- and intermolecular motions in liquid water revealed by two-dimensional terahertz-infrared-visible spectroscopy. Nat. Commun..

[67] Frisch, M. J. b. et al. Gaussian 16, revision A.03. (Gaussian, Inc.: Wallingford, CT, USA, 2016).

[68] Zhao Y, Truhlar DG (2008). The M06 suite of density functionals for main group thermochemistry, thermochemical kinetics, noncovalent interactions, excited states, and transition elements: two new functionals and systematic testing of four M06-class functionals and 12 other functionals. Theor. Chem. Acc..

[69] Wang J, Wolf RM, Caldwell JW, Kollman PA, Case DA (2004). Development and testing of a general amber force field. J. Comput. Chem..

[70] Case, D. A. et al. *Amber 2018* (University of California, 2018).

[71] Krzywinski M (2009). Circos: an information aesthetic for comparative genomics. Genome Res..

[72] Provencher SW (1982). CONTIN: a general purpose constrained regularization program for inverting noisy linear algebraic and integral equations. Comput. Phys. Commun..

[73] Berne, B. J. & Harp, G. D. in *Advances in Chemical Physics* 63−227 (John Wiley & Sons Ltd., 1970).

[74] Day JT (1967). On the numerical solution of linear Volterra integral equations. BIT Numer. Math..

[75] Dai D (2021). Room-temperature DNP NMR spectroscopy of small biological molecules in water, simulations of TEMPOL. Zenodo.

