# Supplementary material for: Room-temperature dynamic nuclear polarization enhanced NMR spectroscopy of small biological molecules in water
Source: Nat Commun. 2021 Nov 25;12:6880. doi: 10.1038/s41467-021-27067-0 (PMC8616939; doi:10.1038/s41467-021-27067-0)
Supplement: Supplementary file 1 — Supplementary Information [file 41467_2021_27067_MOESM1_ESM.pdf]

Supplementary Information

Room-Temperature Dynamic Nuclear Polarization Enhanced NMR Spectroscopy of Small Biological Molecules in Water

Danhua Dai†,[1,2] Xianwei Wang†,[3,4] Yiwei Liu,[3] Xiaoliang Yang,[5,6,] Clemens Glaubitz,[2,7]

Vasyl Denysenkov,[1,2] Xiao He*,[3,8] Thomas Prisner,[1,2] Jiafei Mao*[2,7]

[1.] Institute of Physical and Theoretical Chemistry, Goethe University Frankfurt, 60438 Frankfurt am Main, Germany

[2.] Center for Biomolecular Magnetic Resonance, Goethe University Frankfurt, 60438 Frankfurt am Main, Germany

[3.] Shanghai Engineering Research Center of Molecular Therapeutics and New Drug Development, School of Chemistry and Molecular Engineering, East China Normal University, Shanghai, 200062, China

[4.] College of Science, Zhejiang University of Technology, Hangzhou, Zhejiang, 310023, China

[5.] Jiangsu Key Laboratory of Advanced Organic Materials, School of Chemistry and Chemical Engineering, Nanjing University, Nanjing, 210023, China

[6.] State Key Laboratory of Coordination Chemistry, School of Chemistry and Chemical Engineering, Nanjing University, Nanjing, 210023, China

[7.] Institute of Biophysical Chemistry, Goethe University Frankfurt, 60438 Frankfurt am Main, Germany

[8.] NYU-ECNU Center for Computational Chemistry at NYU Shanghai, Shanghai, 200062, China

† These authors contributed equally to the work

* Corresponding authors. Email addresses: xiaohe@phy.ecnu.edu.cn (X.H., https://orcid.org/0000-0002-4199-8175), j.mao@em.uni-frankfurt.de (J.M., https://orcid.org/0000-0003-1547-325X)

Supplementary Note 1. Brief introduction of ODNP theory for the general audience

ODNP is a result of e-N cross-relaxation. In the case of a spin pair by one electron and one ^{13}C nucleus spins, we consider an energy diagram 2*2 states (electron α and β states and ^{13}C α and β states). The spin dynamics of I (^{13}C) and S (electron) are coupled in this system via cross-relaxation rates R_{IS} and can be described phenomenologically by Solomon equation:

$$\frac{d}{dt}\begin{bmatrix} S_z(t) \\ I_z(t) \end{bmatrix} = \begin{bmatrix} -R_{1S} & -R_{IS} \\ -R_{IS} & -R_{1I} \end{bmatrix} \begin{bmatrix} S_z(t) - S_z(0) \\ I_z(t) - I_z(0) \end{bmatrix}$$

where R_{1S}, R_{1I} and R_{IS} are longitudinal relaxation rates of S-spin, I-spin and the I-S spin pair cross-relaxation rates, respectively.

In a steady state and assuming the electron spin S is only partially saturated by microwave, the explicit form of Solomon equation can be simplified as:

$$\begin{bmatrix} 0 \\ 0 \end{bmatrix} = \begin{bmatrix} -R_{1S} & -R_{IS} \\ -R_{IS} & -R_{1I} \end{bmatrix} \begin{bmatrix} -s \cdot S_z(0) \\ I_z(t) - I_z(0) \end{bmatrix}$$

where s stands for the saturation factor.

The ODNP enhancement (of steady state that assumes adequately long interscan delay) can be obtained from this equation:

$$\varepsilon_{ODNP} = \frac{I_z(t \to \infty)}{I_z(0)} = 1 + s \cdot \frac{R_{IS}}{R_{13C}} \cdot \frac{S_z(0)}{I_z(0)} = 1 - s \cdot \frac{R_{IS}}{R_{13C}} \cdot \left| \frac{\gamma_e}{\gamma_{13C}} \right|$$

where the ^{13}C relaxation rate R_{13C} contains the paramagnetic contributions.

$$R_{13C} = R_{13C}^{dia} + R_{13C}^{para}$$

We than rewrite the above equation as

$$\varepsilon_{ODNP} = 1 - s \cdot \frac{R_{IS}}{R_{13C}^{para}} \cdot \frac{R_{13C}^{para}}{R_{13C}} \cdot \left| \frac{\gamma_e}{\gamma_{13C}} \right|$$

$$\zeta = \frac{R_{IS}}{R_{13C}^{para}} \qquad f = \frac{R_{13C}^{para}}{R_{13C}}$$

where ζ is coupling factor and f is leakage factor. When the ^{13}C T1 relaxation is dictated by the paramagnetic effect, leakage factor f is close to 1. The coupling factor presents the

competition between e-^{13}C cross-relaxation and paramagnetic-enhanced ^{13}C T1 relaxation. The cross-relaxation rate R_{IS} can be further dissected as below:

$$R_{IS}=R_{DQ}-R_{ZQ}=R_{DQ}^{dipolar}-R_{ZQ}^{dipolar}-R_{ZQ}^{scalar}$$

As shown by the scalar spin-spin interaction Hamiltonian, the scalar interaction only drives ZQ relaxation here. Therefore, the ODNP enhancement on ^{13}C is always positive if scalar interaction is dominating.

This equation links the macroscopic relaxation rates with microscopic spin-spin fluctuations. All these cross-relaxations are driven by the fluctuation of respect spin-spin interactions. In the case of rapid isotropic fluctuation, the macroscopic ZQ scalar relaxation can be linked to the microscopic fluctuations as below[1,2]:

$$\widehat{\mathcal{H}}_{IS}^{s}=\frac{1}{2}A(t)(\hat{I}_{+}\hat{S}_{-}+\hat{I}_{-}\hat{S}_{+})$$

$$J_{IS}^{s}(\omega)=\int_{-\infty}^{\infty}\overline{A(t)A(t+\tau)}\,e^{-i\omega\tau}d\tau=\int_{-\infty}^{\infty}|A|^{2}e^{-t/\tau_{s}}\,e^{-i\omega\tau}d\tau=\frac{2\tau_{s}}{\omega^{2}\tau_{s}^{2}+1}$$

$$R_{IS}^{s}=\frac{1}{3\hbar^{2}}J_{IS}^{s}(\omega_{ZQ})=\frac{2}{3}\cdot\left(\frac{A}{\hbar}\right)^{2}\cdot S(S+1)\cdot\frac{\tau_{s}}{\omega_{ZQ}^{2}\tau_{s}^{2}+1}$$

where A stands for the scale of hyperfine interaction, S is the quantum spin number and τ_s is the autocorrelation time of fluctuation of spin-spin scalar interaction. The spectral density function should be in dimension T^{-1}. Here the Lorentzian spectral density function is yielded from the exponential autocorrelation function. These forms can only be held for the idea Ornstein-Uhlenbeck process (**S9**). While the mode of the fluctuation does not match this simplest picture, the modification of the spectral density is required. One simple modification of the above model is so-called "pulse" model[3,4], in which the pulse-composed Poisson process yields an exponential spectral density function:

$$J_{IS}^{s}(\omega)=\frac{4\langle a\rangle^{2}\tau_{w}^{2}}{\tau_{p}}\exp\left(-\omega^{2}\tau_{w}^{2}\right)$$

where $<a>$ is the amplitude of the scalar hyperfine pulse, τ_p^{-1} is the frequency of pulses and τ_w is the width (similar to correlation time) of the pulse.

In our long-living radical-substrate complexes, the mode of the Fermi contact fluctuation driving the scalar cross-relaxation processes is even much more complex. In particular, the autocorrelation function of the fluctuation is not simple exponential forms and the

corresponding spectral density function is very complex. The processes featuring the complex auto-correlation functions can be described more precisely via the autoregressive model (see **S9** below for more details). Here we derive an approximated version to link the spectral density of such complex processes with scalar ZQ relaxation time. In our systems the memory time (10^1 fs) is relative shorter than the Fermi contact fluctuation (spin-density fluctuation, 10^{-1} ps) and both are much shorter than the e-^{13}C cross-relaxation time (10^{-1} to 10^1 s).

$$\tau_{memory} < \tau_{Fermi} \ll T_{IS}^{scalar}$$

So we have taken the scalar relaxation theory in [1] as an approximation and incorporated an "arbitrary" form of spectral density:

$$J_{IS}^{s}(\omega) = \int_{-\infty}^{\infty} \overline{A(t)A(t+\tau)}\, e^{-i\omega\tau} d\tau = \int_{-\infty}^{\infty} \overline{X\rho(t)X\rho(t+\tau)}\, e^{-i\omega\tau} d\tau$$
$$= X^2 \cdot \mathcal{F}[G_\rho(t)] = X^2 \cdot G_\rho(0) \cdot j_\rho(\omega)$$

$$R_{IS}^{s} = \frac{1}{3\hbar^2} J_{IS}^{s}(\omega_{ZQ}) = \frac{1}{3} \cdot \left(\frac{X}{\hbar}\right)^2 \cdot S(S+1) \cdot G_\rho(0) \cdot j_\rho(\omega_{ZQ})$$

where X is the conversion factor from spin density to hyperfine coupling constant, $G_\rho(0)$ is the spin-density autocorrelation function at zero time shift, $j_\rho(\omega_{ZQ})$ is the spectral density at the ZQ frequency.

Supplementary Note 2. Lifetime of indole-TEMPO complex

Here we consider the reaction

$$\text{indole} + \text{TEMPO} \underset{k_{-1}}{\overset{k_1}{\rightleftharpoons}} \text{indole} \cdots \text{TEMPO}$$

The rate constants k_1 and k_{-1} defines equilibrium constant K:

$$K = \frac{k_1}{k_{-1}}$$

The up-limit of the formation rate of indole-TEMPO complex is defined by the diffusion-limited molecular collision in solution:

$$\max(k_1) = k_{Diff} = \frac{2RT}{3\eta}(r_{indole} + r_{TEMPO})(\frac{1}{r_{indole}} + \frac{1}{r_{TEMPO}})$$

in which η is the viscosity of solvent and r presents the molecular radius.

The radius of indole and TEMPO molecules are 3.4 Å[5,6]. Therefore the up-limit of k_1 in CCl_4 at 300 K is:

$$\max(k_1) = \frac{8RT}{3\eta} = 7.4\times10^9\ L \cdot mol^{-1} \cdot s^{-1}$$

The equilibrium constant K was reported in Ref. [7]. Taking this value, the minimal lifetime of indole-TEMPO complex can be obtained:

$$\min(\tau_{complex}) = \frac{1}{\max(k_{-1})} = \frac{\mathrm{K}}{\max(k_1)} = \frac{0.89}{7.4\times10^9} s = 120\ ps$$

Since the equilibrium constant of the indole-TEMPO complex was determined from the paramagnetic effects, the estimated minimal lifetime of indole-TEMPO should be qualified enough for the discussions in this work.

Supplementary Note 3. Binding affinity and lifetime of CCl_4-TEMPONE complex

First, the binding affinity of CCl_4-TEMPONE complex was approximated from the experimental molar-free carbon paramagnetic shift ($\overline{\delta_{para}}$) and carbon Fermi contact hyperfine constant A/h (4.65 MHz) from the previous DFT calculations in Gauss.[8] The Fermi contact shift δ_{con} of CCl_4 in CCl_4-TEMPONE complex can be calculated as:

$$\frac{A}{h}(^{13}C) = \frac{\mu_0\mu_e g_e \mu_N g_{13C}}{3S * h}\rho = 1.124\times10^3\ \rho$$

$$\delta_{con} = \frac{\mu_0\mu_B^2 g_e^2(S+1)}{9kT}\rho = 1.186\times10^5\ \rho$$

where hyperfine coupling constant A/h is in MHz and spin density ρ is in atomic unit (Bohr radius as the distance unit) as also used in the Gaussian program. The contact shift δ_{con} is in ppm and the $\delta_{con} - \rho$ conversion applies ubiquitously for any types of nuclear spins. We take S = ½ and T = 298 K for our case.

Using the above equations, we have the spin density on CCl_4 carbon is $4.14*10^{-3}$ and the corresponding Fermi contact shift would be $1.57*10^3$ ppm. The $\overline{\delta_{para}}$ is only 25.7 ppm,

which is much smaller than the Fermi contact shift, and could contain a dipolar contribution. We can therefore estimate the up-limit of the binding affinity of CCl_4-TEMPONE complex as

$$K \approx \frac{\overline{\delta_{para}}}{\delta_{con}} = 1.6\times10^{-2}M^{-1}$$

Similar to S7, we take the radius of CCl_4 (2.45 Å[9]) and TEMPONE (3.5 Å[10]), we estimate the lifetime of indole-TEMPO complex as 2.1 ps, which is line with the magnitude of previous results[11].

Supplementary Note 4. Brief introduction on the spin density fluctuation - autocorrelation function, spectral density, autoregressive AR model

The spin density trajectory from QM/MM MD trajectory of a single molecular complex *ρ(t)* can be Fourier transformed to a frequency spectral the profile of which is the spectral density function of the random process (Supplementary Figure 4). Alternatively, *the autocorrelation function* $G_\rho(t)$ *and the normalized form* $g_\rho(t)$ *of ρ(t)* can be calculated following:

$$G_\rho(t) = < \rho(t_0)\rho(t_0 + t) >$$

$$g_\rho(t) = \frac{< \rho(t_0)\rho(t_0 + t) >}{< \rho(t_0)\rho(t_0) >}$$

The Fourier transform of autocorrelation function yields spectral density function $J_\rho(t)$ and its normalized form $j_\rho(t)$ (Supplementary Figure 4)

$$J_\rho(\omega) = \mathcal{F}[G_\rho(t)] = \mathcal{F}[g_\rho(t)\cdot G_\rho(0)] = G_\rho(0)\cdot\mathcal{F}[g_\rho(t)] = G_\rho(0)\cdot j_\rho(\omega)$$

$$j_\rho(\omega) = \mathcal{F}[g_\rho(t)]$$

If the distribution of ρ(t) follows a normal distribution, the corresponding process is called a *Gaussian (random) process*. While a Gaussian process shows *an exponential correlation function*, this special process is also called *Ornstein-Uhlenbeck process* that is the most common model of the random fluctuations adapted in the majority of relaxation theories in magnetic resonance field. The Ornstein-Uhlenbeck process is a *Markovian process*, namely the evolution of the current state of the system is solely determined by the current status of the state. A Markovian process has no memory effect ("memoryless"). The spectral density function of an exponential autocorrelation function is a Lorentzian function, which is the

most seen form in NMR relaxation theories. However, the deviation of $\rho(t)$ from the ideal Ornstein-Uhlenbeck process would break down the simplified Lorentzian spectral density function. A simple example is the so-called pulse model[3,4], in which the fluctuation is in the format of a pulse-composed Poisson process, yields a peak at a non-zero frequency. The deviation from the ideal Ornstein-Uhlenbeck can occur more generally than the pulse model. First, if the distribution of $\rho(t)$ in may not follows a symmetric normal distribution, which can be caused by e.g. *an asymmetry barrier* governing the dynamic process. Second, a random process can possess a memory effect e.g. in the presence of restraints defined by the previous steps or under the "slow" impacts that require certain time to propagate to the interested site. Such behaviors often emerge in highly coupled systems. A random process with memory effect is called a *non-Markovian process*. To describe this *memory effect*, the so-called *memory function $K_g(t)$* can be derived following:

$$\frac{\partial}{\partial t} G_\rho(t) = -\int_0^t K_g(\tau)\, g_\rho(t-\tau)\, d\tau$$

Here $K_g(t)$ is also sometimes called *memory kernel*. The solution $K_g(t)$ of the above Volterra integral equation for an exponential $g_\rho(t)$ is a δ-function, which indicates a zero-memory effect. While $g_\rho(t)$ describes a non-Markovian process, the corresponding $K_\rho(t)$ shows a finite decay. (Supplementary Figure 4)

In a highly simplified picture, a random Ornstein-Uhlenbeck process drives the spin relaxation when the correlation time of the random process approaches the characteristic frequency (e.g. e-^{13}C ZQ frequency for the scalar cross-relaxation) of the specific spin Hamiltonian responsive for the relaxation. In this picture, *the random process (e.g. a molecular motion) opens the relaxation channel for dissipating the spin energy to the environment (or so-called "lattice")*. The stochastic feature often indicates that such a process is coupled to a large degree of freedom and therefore ensures the presence of a lattice to which the spin energy can dissipate efficiently. Here, the stochastic process can be viewed as a "superposition" of a large number of individual processes that seems coupled due to the complexity (high degrees of freedom) of the system ("lattice").

In our QM/MM simulations, peaks appear in low THz frequency regime that also hits e-^{13}C ZQ frequency. Conceivably the local coupling of spin interactions and the low THz motion takes place. Different from the IR modes that are often originated from highly localized molecular motions, THz modes arise commonly from the collective motions involve higher degrees of freedom IR motions. The presence of THz oscillation features at various molecular interfaces (indole/TEMPO, TEMPO/CCl_4) suggests the further extension of the coupling network.(Supplementary Figure 7m-a) In our system, the coupling of certain low-

THz modes to the environment is likely present as indicated by the significant breath of the modes that are beyond the resolution of the Fourier transform.(Supplementary Figure 7)

To resolve the complex pattern of autocorrelation functions derived from our QM/MM MD simulations, we have turned to the time series analysis with a rather generally used model called autoregressive model (AR). AR model describes the time evolution of spin density $\rho(t)$ following the hypothesis:

$$\rho(t_n) = \sigma(t_n) + \sum_{i=1}^{p} \theta_i \, \rho(t_{n-i})$$

where $\rho(t_n)$ is related to p previous steps with coefficient θ_1(previous point) to θ_p(previous p^{th} point) and $\sigma(t)$ is the random noise with zero average and variance σ^2. In a discrete form such as the QM/MM MD simulation, an Ornstein-Uhlenbeck process can be described by memoryless AR(1) model. AR of higher orders contains a memory effect.

We can apply Z-transform to AR(p) model:

$$P(z)(1 + \sum_{i=1}^{p} \theta_i \, z^{-k}) = E(z)$$

$$z = \exp\,(i\omega T_s)$$

where T_s is the sampling period, and ω is frequency.

The spectral density function (square root of power spectrum) can be expressed in the following form:

$$G(\omega) = \left| \frac{\sigma}{1 + \sum_{i=1}^{p} \theta_i \exp\,(-ip\omega T_s)} \right|$$

The AR(p>1) models show three unique characteristics relevant to our data analysis. First, the autocorrelation function of an AR(p>1) model shows both an initial decay and the sinusoidal waves. Second, the spectral density function (or its squared power spectrum) of AR(p>1) model show p/2 peaks. Third, the damping factor D, frequency Ω and phase φ of the sinusoidal waves in power spectrum are determined by the factors θ_i. For example, in a simple AR(2) model, we have[12]:

$$D = \sqrt{-\theta_2}\,, \quad \Omega = cos^{-1}\left(\frac{\theta_1}{\sqrt{-2\theta_2}}\right), \quad \varphi = tan^{-1}\left(\frac{1-\theta_2}{1+\theta_2}\right)$$

Supplementary Note 5. Estimation of zero-quantum e-^{13}C scalar cross-relaxation rates from QM/MM MD simulation

In indole-TEMPO complex $\tau_{memory} < \tau_{Fermi} \ll T_{ZQ}$, so we use the conventional relaxation theory of the isotropic fluctuation of spin-spin scalar coupling[1] as an approximation for evaluating the impact of spin density fluctuation obtained from QM/MM MD in driving e-^{13}C ZQ cross-relaxation. We have

$$R_{ZQ} = \left(\frac{2\mu_0\mu_e g_e \mu_N g_{13C}}{3\hbar}\right)^2 \cdot G(0) \cdot j(\omega_{ZQ})$$

where the first term scales the spin density to Fermi hyperfine constant as defined $A/\hbar$ (= $2\pi A/h$). The $G(0) * j(\omega)$ is essentially equal to the spectral density of spectral density function $J(\omega)$ of unnormalized spin density autocorrelation function $G_\rho(t)$. The ZQ frequency ω_{ZQ} is equal to $2\pi * f_{ZQ}$ (1.652 THz). The spectral density functions presented in Supplementary Figure 7 are in the unit of fs (10^{-15} s). The estimated ZQ scalar cross-relaxation rates are listed in Supplementary Table 6. These reates are mostly in the order of magnitude of 10^{-1} to 10^{1} s^{-1}, which agrees qualitatively with the observed DNP enhancement factors.

Supplementary Note 6. The relevance of paramagnetic NMR in DNP inspired by a Google search

By taking the molecules “halogenated solvent” and “radical” investigated in a previous seminal ODNP work[8] as the key words, a Google search returned a collection of EPR and computational chemistry studies on these catalogues of molecules. Interestingly this search, likely “biased” by the personal search history, also suggested a paramagnetic NMR work on relevant molecular systems,[13] which immediately enlightened us about an overlooked link between paramagnetic NMR and ODNP spectroscopy.

Supplementary Discussion. Additional factors that may complicates the derivation of ODNP enhancement from paramagnetic NMR and QM/MM MD results

It appears that a simple correlation between $\overline{\delta_{para}}$, the spectral density function of Fermi contact fluctuation and the ODNP enhancement is not straightforward (Supplementary Table 6), which is likely due to multiple factors. The $\overline{\delta_{para}}$ parameter contains also the dipolar contribution and is impacted by macroscopic magnetic susceptibility, therefore only provides a qualitative rather than the quantitative measure of spin density. The frequency-resolution of the spectral density function derived from our QM/MM MD trajectory is still limited by the total duration (10 ps) of the simulation. A follow-up *ab initio* computational

study aiming a trajectory beyond 100 ps is currently underway. Our first glimpse into the more extended 20-ps trajectory shows that the oscillations at 10^2 fs time scale indeed persist (Supplementary Figure 5t, S6t). Beside the complexities in $\overline{\delta_{para}}$ and computed spectral density, the observed ODNP enhancement also contains contributions from the other hyperpolarization transfer pathways including the direct dipolar e-^{13}C ODNP and the indirect e-^{1}H-^{13}C polarization transfer. The direct dipolar e-^{13}C ODNP yields negative ^{13}C enhancement that could neutralize partially the positive scalar ODNP enhancement. This contribution is especially profound on the bridge carbons in indole and the carboxylate carbons in amino acids. Similar to the scalar e-N interaction, the dipolar e-N crosstalk in a long-living radical-substrate complex also presents rather complex dynamic features, not to mention the breaking-down of the point-dipole approximation in the closely engaged radical-substrate complexes with delocalized spin densities (Supplementary Figure 11j-p). We have also observed in general the negative ^{1}H enhancement in our systems (Supplementary Figure 1e-f, Supplementary Table 3-S4). In fast-tumbling small molecules, the negatively enhanced ^{1}H polarization could interconvert with the positive ^{13}C hyperpolarization via ^{1}H-^{13}C heteronuclear NOE (hNOE) effect (Supplementary Figure 11u-v).[14-16] All the abovementioned mechanistic complexities perplex the quantitative prediction of ODNP enhancement, which consolidates the value of $\overline{\delta_{para}}$ as a qualitative but highly practical indicator of scalar ODNP performance.

Supplementary Table 1. ODNP NMR samples used in this work

Target molecule	Target concentration (M)	Radical*	Solvent
Indole (U-^{13}C)	2.0	TEMPO	CCl_4
Indole (2-^{13}C)	2.0	TEMPO	CCl_4
$^{13}CCl_4$	≈ 20	TEMPONE	$^{13}CCl_4$
imidazole (2-^{13}C, U-^{15}N)	1.0	TEMPO	$CDCl_3$
imidazole (2-^{13}C, U-^{15}N)	2.0 (pH* = 11.0)	TEMPOL	D_2O
glucose (U-^{13}C)	2.0 (pH = 7.3)	TEMPOL	H_2O
glycine (U-^{13}C)	2.0 (pH = 6.1)	TEMPOL	H_2O
alanine (U-^{13}C)	1.4 (pH = 6.0)	TEMPOL	H_2O
serine (U-^{13}C)	2.0 (pH = 5.7)	TEMPOL	H_2O
proline (U-^{13}C)	2.0 (pH = 6.2)	TEMPOL	H_2O

* radical concentration was 100 mM for all samples

Supplementary Table 2. Summary of ODNP NMR spectra of indole, imidazole and CCl_4 acquired on HC probehead

compound	solvent	radical	site	chemical shift* (ppm, - mw)	chemical shift* (ppm, + mw)	$^1J_{CH}$ (Hz)	$^1J_{CC}$ (Hz)	DNP Enhancement
**indole**	**CCl_4**	**TEMPO**	C2	124.6	123.3	181	67	14.1 ± 2.5 ($^{13}C_2$)<br>26.2± 2.4 (U-^{13}C)
			C3	102.2	101.4	170	62	11.6 ± 1.1
			C3a	127.0	127.0†	-	-	-1.8 ± 0.5
			C4/5/6	119.7	119.5	n.r.‡	n.r.‡	5.4 ± 0.2
			C7	111.0	109.9	157	62	20.7 ± 2.5
			C7a	134.5	135.0	-	-	-2.1 ± 0.5
			H_N¶	7.23	7.04	-	-	-9.5 ± 0.0
			H2¶	6.61	6.31	n.r.‡	-	-1.5 ± 0.1
			H3¶	6.41	6.17	n.r.‡	-	-8.7 ± 0.0
			H4¶	7.57	7.30	n.r.‡	-	-8.9 ± 0.1
			H5/6¶	7.07	6.83	n.r.‡	-	-10.9 ± 0.0
			H7¶	6.87	6.60	n.r.‡	-	-11.8 ± 0.0
**CCl_4**	**CCl_4**	**TEMPONE**	C	97.9	91.7	-	-	92.4 ± 19.4
**imidazole§**	**$CDCl_3$**	**TEMPO**	H2	7.86	7.57	n.r.‡	-	-5.2 ± 0.4
			H4/5	7.30	7.02	n.r.‡	-	-4.0 ± 0.2
**imidazole§**	**D_2O**	**TEMPOL**	C2	-	136.2	n.r.‡	-	> 0 (low S/N)
			H2	7.70	7.36	n.r.‡	-	-12.3 ± 4.0
			H4/5	7.33	7.02	n.r.‡	-	-18.7 ± 1.7
			HDO	4.70	3.34	-	-	-39.2 ± 2.4

* all chemical shifts are referenced indirectly to TMS
† this peak is assigned tentatively
‡ not resolved (n.r.)
¶ values extracted from spectral deconvolution
§ no stable enhancement values can be obtained due to the severe sample heating

Supplementary Table 3. Summary of ODNP NMR spectra of water-soluble compounds acquired on FP probehead

compound	solvent	radical	site	chemical shift* (ppm, - mw)	chemical shift* (ppm, + mw)	$^1J_{CH}$ (Hz)	$^1J_{CC}$ (Hz)	DNP Enhancement
**imidazole**	**D_2O**	**TEMPOL**	C2	136.2	136.0	n.r.‡	n.r.‡	50 ± 10
			H2/4/5	7.12 (broad)	7.64	n.r.‡	n.r.‡	-5.0 ± 0.3
			HDO	4.70	5.18	-	-	-9.5 ± 0.2
**glucose**	**H_2O**	**TEMPOL**	C1 (β)	96.1	95.9	154	n.r.‡	5.9 ± 1.9
			C1 (α)	92.2	92.0	156	n.r.‡	6.9 ± 2.2
			C3 (β)	76.0	75.9	n.r.‡	59	
			C2 (β)	74.2	74.2	n.r.‡	59	2.2 ± 0.4 (C2/3/4/5)
			C3 (α)	73.0	73.1	n.r.‡	50	
			C2 (α)/C4 (α+β)	71.5	71.5	n.r.‡	65	
			C5(α+β)	69.8	69.9	n.r.‡	74	
			C6 (α+β)	61.1	61.1	136	n.r.‡	6.8 ± 1.8
			$H_{glucose}$ + H_2O	4.70	4.65	n.r.‡	-	-8.3 ± 0.2
**glycine**	**H_2O**	**TEMPOL**	Cα	41.8	41.9	141	n.r.‡	10.7 ± 1.7
			C’	172.5	n.r.‡	-	n.r.‡	ca. -1†
			Hα	3.16¶	3.22¶	n.r.‡	n.r.‡	10.0 ± 0.1¶
			H_2O	4.70	5.02	n.r.‡	-	-9.8 ± 0.1
**alanine**	**H_2O**	**TEMPOL**	Cα	50.8	50.8	134	n.r.‡	6.0 ± 1.6
			Cβ	16.7	16.4	131	n.r.‡	6.1 ± 2.2
			C’	175.9	n.r.‡	-	n.r.‡	ca. -1†
			Hβ	1.19	n.r.‡	n.r.‡	n.r.‡	n.r.‡
			Hα + H_2O	4.70	4.48	n.r.‡	n.r.‡	-10.2 ± 0.1
**serine**	**H_2O**	**TEMPOL**	Cα	56.5	56.2	155	n.r.‡	6.0 ± 0.1
			Cβ	60.3	60.0	145	n.r.‡	6.5 ± 0.1
			C’	172.5	n.r.‡	-	n.r.‡	ca. -1†
			H_{serine} + H_2O	4.70	4.03	n.r.‡	n.r.‡	-7.0 ± 0.1

Supplementary Table 3. continue

compound	solvent	radical	site	chemical shift* (ppm, - mw)	chemical shift* (ppm, + mw)	$^1J_{CH}$ (Hz)	$^1J_{CC}$ (Hz)	DNP Enhancement
**proline**	**H_2O**	**TEMPOL**	Cα	61.3	61.5	144	n.r.‡	4.5 ± 0.5
			Cδ	46.3	46.3	145	n.r.‡	10.5 ± 0.8
			Cβ	29.1	29.0	128	n.r.‡	6.9 ± 0.7
			Cγ	23.8	23.8	133	n.r.‡	8.2 ± 0.7
			C’	174.5	n.r.‡	-	n.r.‡	ca. -1†
			Hβ2/γ	1.78	1.98	n.r.‡	n.r.‡	-2.9 ± 0.2
			$H_{\alpha/\beta1/\delta}$ + H_2O	4.70	3.39	n.r.‡	n.r.‡	-7.0 ± 0.1

* all chemical shifts are referenced indirectly to TMS

† this value is estimated by the disappearance of C’ signal on ODNP NMR spectrum

‡ not resolved (n.r.)

¶ values extracted from spectral deconvolution

Supplementary Table 4. Summary of paramagnetic shifts and ODNP enhancements*

compound	solvent	radical	site	Molar-free paramagnetic chemical shift (ppm)	DNP Enhancement
**indole**	**CCl_4**	**TEMPO**	C2	16.21 ± 0.09	14.1 ± 2.5 ($^{13}C_2$)<br>26.2± 2.4 (U-^{13}C)
			C3	14.65 ± 0.07	11.6 ± 1.1
			C3a	11.66 ± 0.07	-1.8 ± 0.5
			C4/5/6	8.17 ± 0.38	5.4 ± 0.2
			C7	14.42 ± 0.09	20.7 ± 2.5
			C7a	10.19 ± 0.07	-2.1 ± 0.5
**CCl_4**	**CCl_4**	**TEMPONE**	C	25.69 ± 0.07	92.4 ± 19.4<br>(430 ± 50)[†]
**CBr_4**	**CCl_4**	**TEMPONE**	C	120.4 ± 0.7	128.9 ± 12.9[‡]<br>(600 ± 60)[†]
**$CHCl_3$**	**$CHCl_3$**	**TEMPONE**	C	46.13 ± 0.63	68.8 ± 12.9[‡]<br>(320 ± 60)[†]
**diethyl malonate**	**diethyl malonate**	**TEMPONE**	C2	13.38 ± 0.11	6.4 ± 2.1[‡] (30 ± 10)[†]
**ethyl acetoacetate**	**ethyl acetoacetate**	**TEMPONE**	C2	11.36 ± 0.09	3.9 ± 0.2[‡] (18 ± 1)[†]
			C4	10.85 ± 0.08	2.8 ± 0.4[‡] (13 ± 2)[†]
**pyruvic acid**	**pyruvic acid**	**TEMPONE**	C3	6.28 ± 0.15	2.8 ± 0.4[‡] (13 ± 2)[†]
**imidazole**	**D_2O**	**TEMPOL**	C2	4.93 ± 0.02	50 ± 10
**glucose**	**H_2O**	**TEMPOL**	C1 (β)	1.86 ± 0.02	5.9 ± 1.9
			C1 (α)	2.05 ± 0.02	6.9 ± 2.2
			C3 (β)	1.78 ± 0.02	
			C2 (β)	1.43 ± 0.02	2.2 ± 0.4 (C2/3/4/5)
			C3 (α)	1.27 ± 0.01	
			C2 (α)/C4 (α+β)	1.87 ± 0.02	
			C5(α+β)	1.47 ± 0.02	
			C6 (α+β)	3.06 ± 0.02	6.8 ± 1.8

Supplementary Table 4. Continue

compound	solvent	radical	site	Molar-free paramagnetic chemical shift (ppm)	DNP Enhancement
**glycine**	**H_2O**	**TEMPOL**	Cα	2.98 ± 0.03	10.7 ± 1.7
			C’	0.68 ± 0.01	ca. -1
**alanine**	**H_2O**	**TEMPOL**	Cα	1.93 ± 0.02	6.0 ± 1.6
			Cβ	3.27 ± 0.02	6.1 ± 2.2
			C’	0.68 ± 0.01	ca. -1
**serine**	**H_2O**	**TEMPOL**	Cα	1.83 ± 0.01	6.0 ± 0.1
			Cβ	2.43 ± 0.01	6.5 ± 0.1
			C’	0.68 ± 0.01	ca. -1
**proline**	**H_2O**	**TEMPOL**	Cα	2.11 ± 0.04	4.5 ± 0.5
			Cδ	3.52 ± 0.04	10.5 ± 0.8
			Cβ	3.06 ± 0.04	6.9 ± 0.7
			Cγ	3.59 ± 0.05	8.2 ± 0.7
			C’	0.27 ± 0.01	ca. -1

* DNP enhancements in organic solvents (white background) or water (gray background) were measured on HC probehead.or FP probehead respectively. All enhancement values are determined at 9.4 T.

† the DNP enhancement in parenthesis was reported in Ref. [1].

‡ the DNP enhancement value was scaled from the results reported in Ref. [1] via the DNP enhancements determined on CCl_4.

Supplementary Table 5. Summary of spin density and hyperfine coupling of TEMPOL-amino acid complexes by DFT calculations

substrate	binding site	H-bond length (Å)	H-bond angle (°)	ΔΔG† (kJ/mol)	spin density |ρ|*10⁴ (atomic unit)					
					N	C'	Cα	Cβ	Cγ	Cδ
**glycine**	NH_2	2.092	158.0	0	68.6	23.2	27.7	-	-	-
	Cα	2.400	118.5	7.65	1.26	7.44	52.8	-	-	-
**alanine**	NH_2	2.076	164.8	0.00	71.8	1.33	6.88	12.6	-	-
	Cα	2.326	131.3	7.67	0.33	2.95	13.3	21.9	-	-
	Cβ (CH_3)	2.447	165.5	8.74	1.36	0.18	1.19	20.3	-	-
**serine**	NH_2	2.098	155.9	0.00	96.4	0.09	2.00	2.30	-	-
	Cα	2.294	125.0	0.18	1.71	5.5	9.09	1.40	-	-
	Cβ	2.452	163.0	3.37	4.24	0.64	3.88	36.9	-	-
	OH	1.815	161.6	-15.7	0.22	0.24	5.90	11.1	-	-
**proline**	NH	2.125	162.5	0.00	15.7	4.22	2.62	0.74	0.59	1.43
	Cα	2.307	137.6	3.82	0.27	0.05	37.6	13.22	1.26	1.20
	Cβ	2.453 (Hβ1)	137.8	3.42	0.76	29.5	6.42	70.03	1.34	7.14
		2.381 (Hβ2)	161.7	0.76	1.13	0.52	5.39	45.2	44.7	2.33
	Cγ	3.018 (Hγ1)*	93.9	3.84	0.50	7.48	4.02	60.9	3.15	9.80
		2.640 (Hγ2)	153.9	4.96	3.01	0.31	6.41	1.30	51.9	14.4
	Cδ	2.661 (Hδ1)	161.6	8.31	0.26	1.35	0.27	1.18	0.41	1.15
		2.384 (Hδ2)	157.6	2.84	0.37	0.16	0.26	1.26	0.33	22.9

† referenced to the energy of TEMPO-substrate(NH) complex

* the optimization consistently yields a structure similar to the Hβ1 complex as indicated by the much higher spin density on Cβ than Cγ

Supplementary Table 6. Summary of the mechanistic analysis of indole ODNP behaviors based on paramagnetic NMR and QM/MM MD

site	molar-free $\overline{\delta_{para}}$ (ppm)	Average spin density $\lvert\rho\rvert*10^4$ (atomic unit)	average A/h (MHz)	G(0)*10^4	$j_{ZQ}*10^2$ † (fs)	$Rs,_{ZQ}(s^{-1})$ ‡ QM/MM MD	^{13}C T1 (- radical)	^{13}C T1 (+ radical)	leakage factor
C2	16.21 ± 0.09	3.26	0.367	4.55	2.4	0.77	2.63 ± 0.07	0.85 ± 0.08	0.68 ± 0.05
C3	14.65 ± 0.07	0.664	$7.46*10^{-2}$	12.0	1.9	1.1	3.01 ± 0.09	1.19 ± 0.03	0.60 ± 0.00
C3a	11.66 ± 0.07	1.35	0.152	5.45	5.2	1.4	15.3 ± 1.0	1.05 ± 0.03	0.93 ± 0.04
C4/5/6	8.17 ± 0.38	5.83 (C4)	0.655 (C4)	2.05 (C4)	2.1	0.26 (C4)	2.62 ± 0.08 (C4)	1.46 ± 0.03 (C4)	0.44 ± 0.00 (C4)
		32.3 (C4)	3.63 (C5)	1.48 (C4)		0.098 (C5)	2.67 ± 0.05 (C5)	1.28 ± 0.02 (C5)	0.52 ± 0.00 (C5)
		8.19 (C4)	0.921 (C6)	12.6 (C4)		1.4 (C6)	2.34 ± 0.10 (C6)	1.68 ± 0.04 (C6)	0.28 ± 0.01 (C6)
C7	14.42 ± 0.09	1.83	0.205	785	2.2	85	2.62 ± 0.08	1.37 ± 0.04	0.48 ± 0.00
C7a	10.19 ± 0.07	0.579	$6.51*10^{-2}$	98.3	2.4	12	14.8 ± 1.0	1.07 ± 0.02	0.93 ± 0.05

† j_{ZQ} presents the "spectral density" at ω_{ZQ} on the spectral density function Fourier transformed from the normalized spin density autocorrelation function.
$\omega_{ZQ} = \omega_e - \omega_{13C}$ = (263-0.1)*2π GHz = 1.652 THz
‡ $Rs,_{ZQ}$ is the scalar coupling-driven relaxation

site	molar-free $\overline{\delta_{para}}$ (ppm)	$Rs_{,ZQ}$ (s^{-1}) QM/MM MD	^{13}C T1 (+ radical)	leakage factor	coupling factor	R_{DQ} - R_{ZQ} (s^{-1}) (back-fitted)	ODNP enhancement
C2	16.21 ± 0.09	0.77	0.85 ± 0.08	0.68	-0.013 ($^{13}C_2$)<br>-0.024 (U-^{13}C)	-0.015 ($^{13}C_2$)<br>-0.029 (U-^{13}C)	14.1 ± 2.5 ($^{13}C_2$)<br>26.2± 2.4 (U-^{13}C)
C3	14.65 ± 0.07	1.1	1.19 ± 0.03	0.60	-0.012	-0.010	11.6 ± 1.1
C3a	11.66 ± 0.07	1.4	1.05 ± 0.03	0.93	0.0020	0.002	-1.8 ± 0.5
C4/5/6	8.17 ± 0.38	0.26 (C4)<br>0.098 (C5)<br>1.4 (C6)	1.46 ± 0.03 (C4)<br>1.28 ± 0.02 (C5)<br>1.68 ± 0.04 (C6)	0.44 (C4)<br>0.52 (C5)<br>0.28 (C6)	-0.0065(C4)<br>-0.0056 (C5)<br>-0.010 (C6)	-0.005 (C4)<br>-0.004 (C5)<br>-0.006 (C6)	5.4 ± 0.2
C7	14.42 ± 0.09	85	1.37 ± 0.04	0.48	-0.027	-0.020	20.7 ± 2.5
C7a	10.19 ± 0.07	12	1.07 ± 0.02	0.93	0.0022	0.002	-2.1 ± 0.5

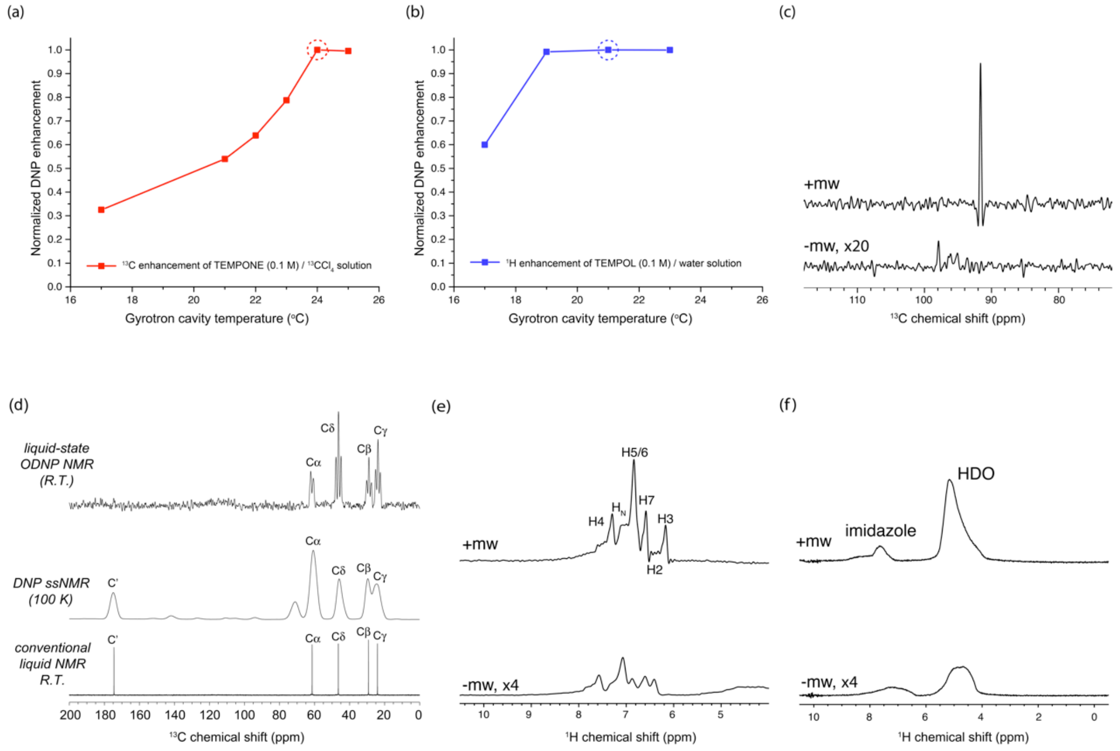

Supplementary Figure 1. ODNP NMR spectra of various compounds in organic solvent or water acquired on a 9.4 T spectrometer. (a-b) The initial search for the proper DNP working condition was performed by scanning the gyrotron cavity temperature and monitoring the signal intensity of the DNP enhanced signals of particular standard samples. (c) The ODNP enhanced ^{13}C NMR spectrum of $^{13}CCl_4$. The microwave (mw) off spectrum was accumulated with 512 scans and the mw on spectrum was acquired with 1 scan. (d) A comparison of the ODNP-enhanced R.T. liquid-state ^{13}C NMR spectrum, DNP-enhanced MAS ssNMR ^{13}C spectrum (CP-based, 110 K) and the conventional ^{1}H-decoupled liquid-state ^{13}C NMR spectra of proline acquired at 9.4 T. (e-f) ODNP-enhanced ^{1}H NMR spectra of indole and imidazole. For the spectra shown in (j), 128 and 8k scans were accumulated for the mw-on and mw-off conditions. For the spectra shown in (k), 64 and 2k scans were accumulated for the mw-on and mw-off conditions. The phase of the DNP-enhanced ^{1}H spectra are inverted here. Here, all the paired mw-on and mw-off spectra were scaled by the number of scans so that the enhancement-factors can be visualized directly despite the distinct noise level due to the very different numbers of scans used for mw-on and mw-off spectra.

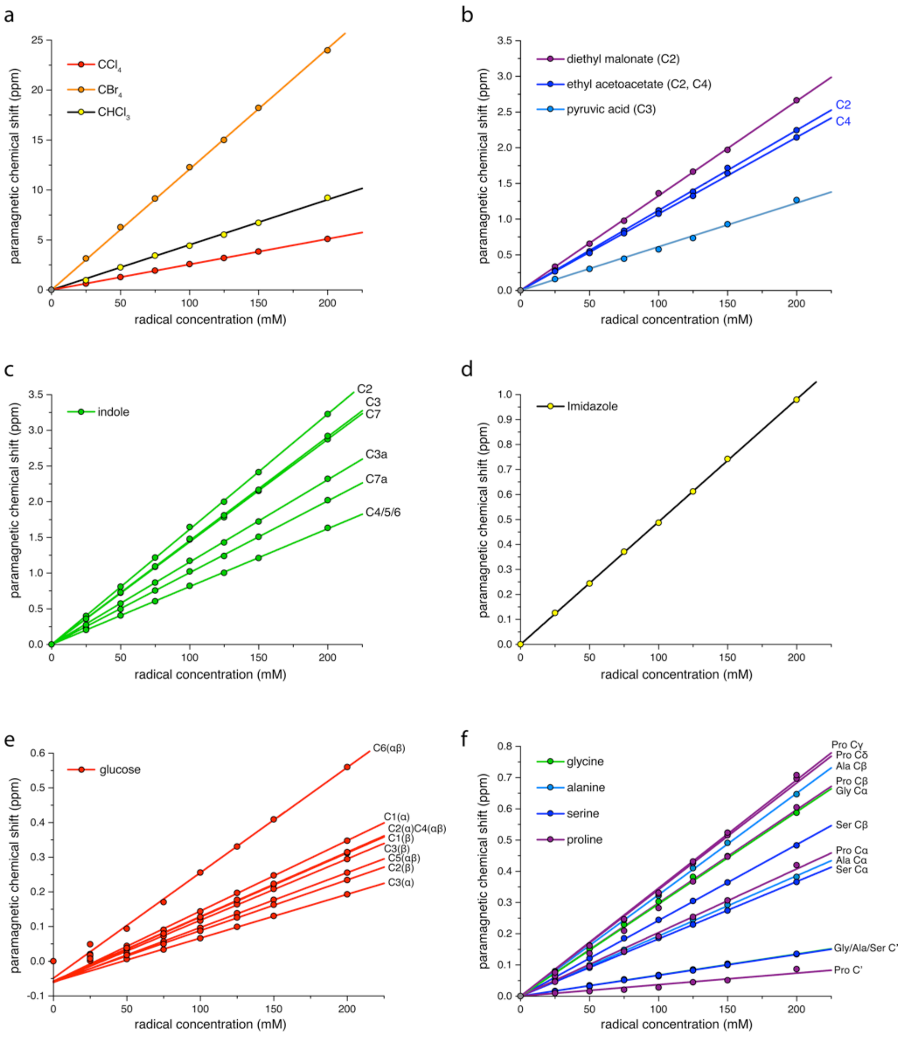

Supplementary Figure 2. Fitting of ^{13}C chemical shifts in the presence of radical at various concentrations. The molar free paramagnetic chemical shift $\overline{\delta_{para}}$ was obtained as the slope of the linear fitting of observed ^{13}C chemical shift versus the concentration of radical. The data shown in a-c were collected in organic solvents and the data in d-f were obtained in water.

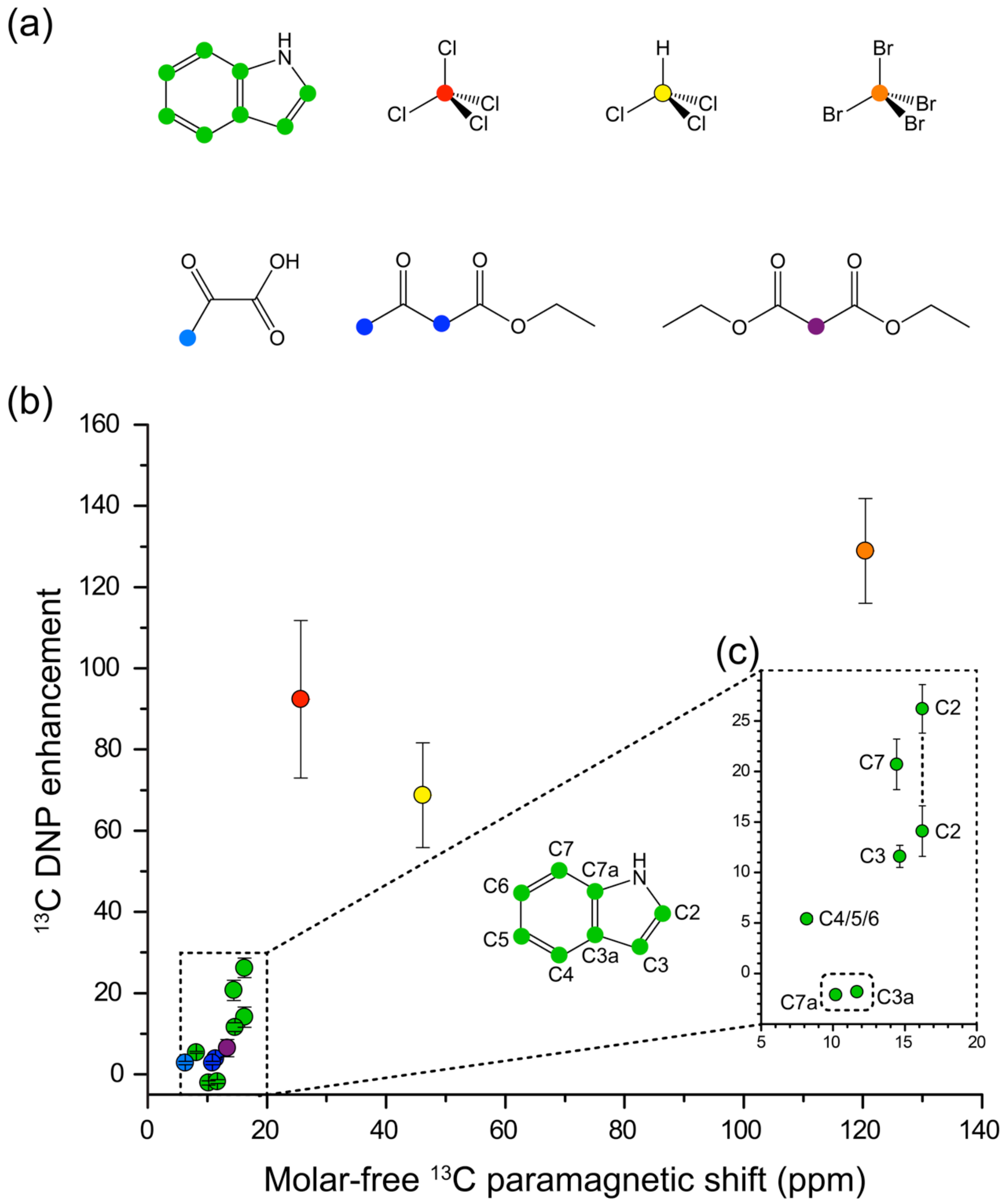

Supplementary Figure 3. ODNP ^{13}C NMR enhancement and the molar free ^{13}C paramagnetic NMR shift of various organic compounds in organic solvents. (a) The structures of the compounds. (b) A trend is visible though more deviated than that shown in Fig. 2f. (c) Data points of different ^{13}C sites in indole. Data obtained on different molecules are shown in various colors. The error of $\overline{\delta_{para}}$ is defined as fitting error or standard deviation of $\overline{\delta_{para}}$ values on carbons showing overlapping signals on ODNP spectra. The error of ODNP enhancement is determined from signal-to-noise ratio. More details about the error calculations can be found in 'Methods'.

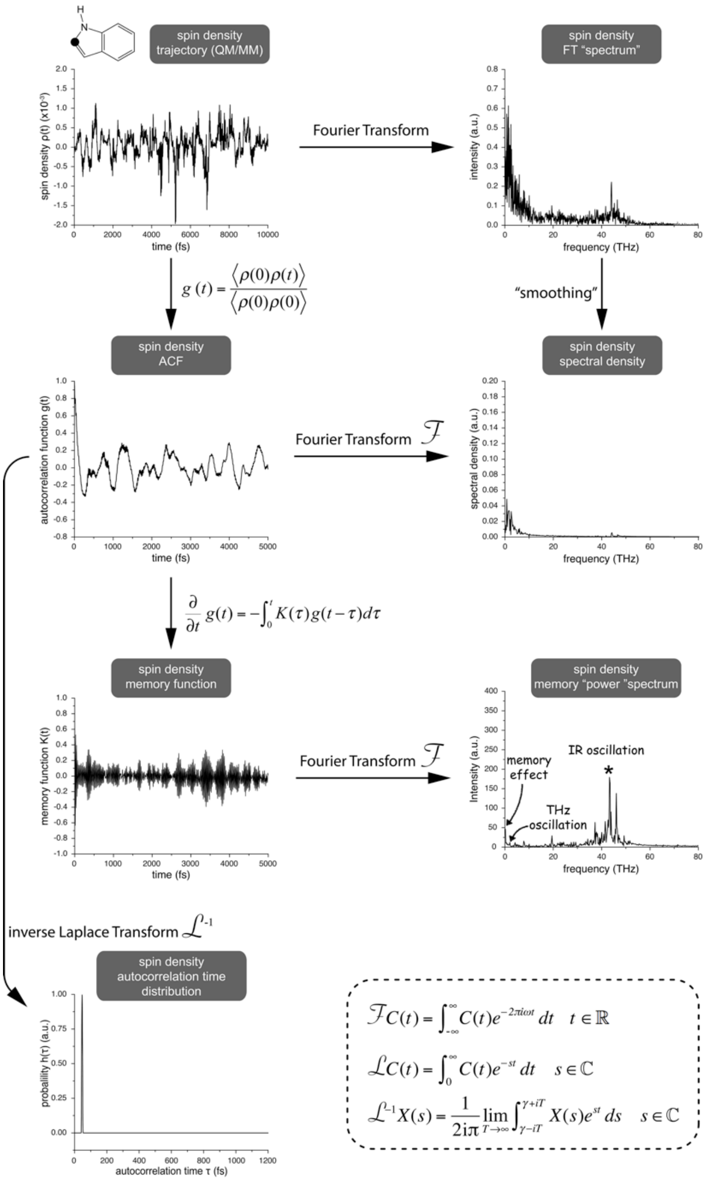

Supplementary Figure 4. Flow chart of the data analysis procedure of QM/MM MD trajectory. The detailed theoretical and mathematical grounds can be found in **S9**. The autocorrelation function $g(t)$ was computed from the trajectory data $\rho(t)$. The Fourier transform of normalized $g(t)$ gives rise to the spectral density function $j(\omega)$. The spectral density function also presents the profile of the frequency spectrum obtained by the direct Fourier transform of the trajectory time-series $\rho(t)$. The time constant of initial decay of $g(t)$ can be estimated by performing the inverse Laplace transform of $g(t)$. This fits the decay to a distribution of rates of the highly simplified exponential decay, therefore can only be taken as an estimation of the true decay kinetics of $g(t)$. The memory function $K(t)$ was further computed from $g(t)$ and the Fourier transform of $K(t)$ yields an amplitude spectrum of $K(t)$. The fast oscillations in $\rho(t)$ can be visualized in $g(t)$ and be better detected in $j(\omega)$, $K(t)$ and the Fourier transformed $K(t)$.

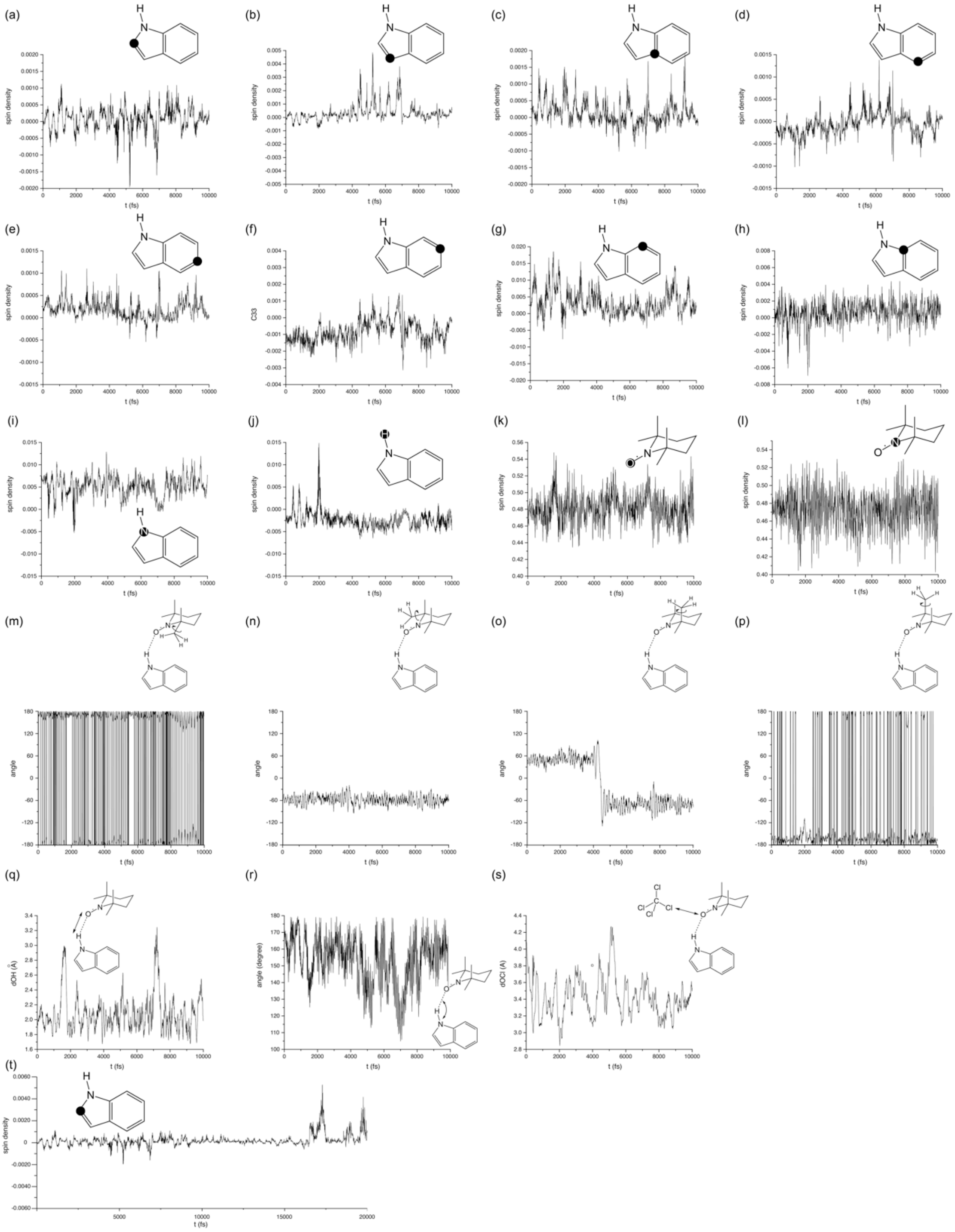

Supplementary Figure 5. Spin density (a-l) and geometry trajectories (m-s) extracted from the QM/MM MD simulation of indole-TEMPO complex in explicit CCl_4 solvent. The time duration of all trajectories is 10 ps. The spin density fluctuations at various atoms on indole and TEMPO are shown in (a-l). The rotation of TEMPO methyl groups are presented by the H(methyl)-C(methyl)-C(TEMPO ring)-N(TEMPO) torsion angle in (m-p). The fluctuations of indole-TEMPO H-bond length and the TEMPO(oxygen)-Cl(chlorine) distance are shown in (q) and (s), while the dynamics of the intermolecular H-bond angle in indole-TEMPO complex is shown in (r). For indole C2, a longer 20 ps spin density trajectory is presented in (t).

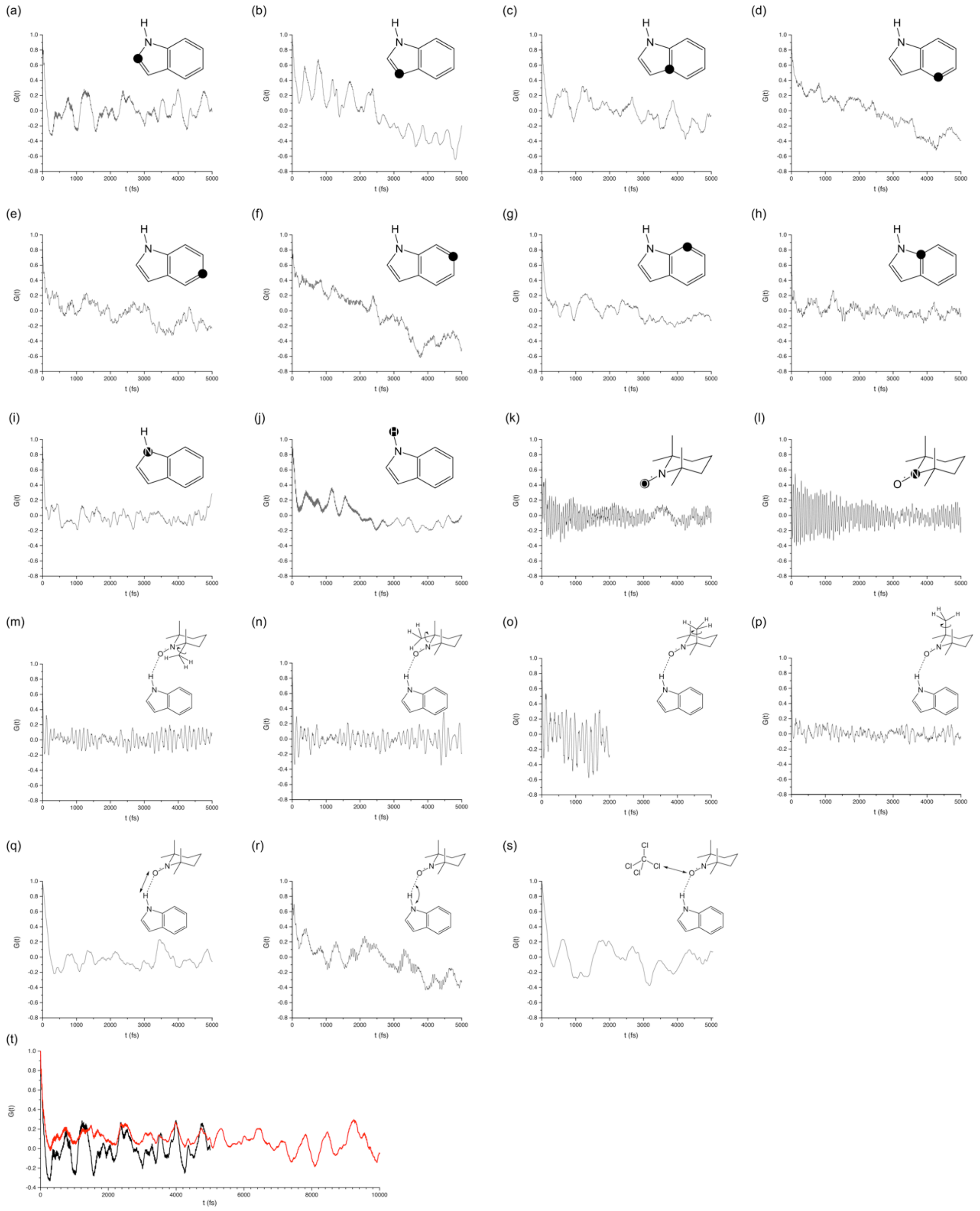

Supplementary Figure 6. Spin density (a-l) and geometry (m-s) autocorrelation functions $G(t)$ derived from the QM/MM MD simulation of indole-TEMPO complex in explicit CCl_4 solvent. The time duration is 5 ps. The spin density autocorrelation functions at various atoms on indole and TEMPO are shown in (a-l). The autocorrelation functions of the TEMPO methyl rotation process are presented in (m-p). The autocorrelation function shown in (o) is truncated due to the "intermittent" jumping in the corresponding trajectory. (Supplementary Figure 3o) The autocorrelation functions of indole-TEMPO H-bond length, indole-TEMPO H-bond angle and TEMPO(oxygen)-Cl(chlorine) distance are shown in (q-s). All autocorrelation functions feature

an initial decay compounded with oscillations components. The spin autocorrelation functions of indole atoms (a-j) and the autocorrelation functions of intermolecular geometry (q-s) show clearly the sub-ps oscillations. The spin density autocorrelation functions of TEMPO NO moiety (k-l) show more significant fs oscillations. The methyl rotation autocorrelation functions also show some fs oscillations. The spin density ACF of indole C2 derived from the extended 20 ps trajectory (Supplementary Figure 4t) is presented in (t). The major features of the ACF pattern, namely the initial decay and the oscillations, remains on this longer ACF.

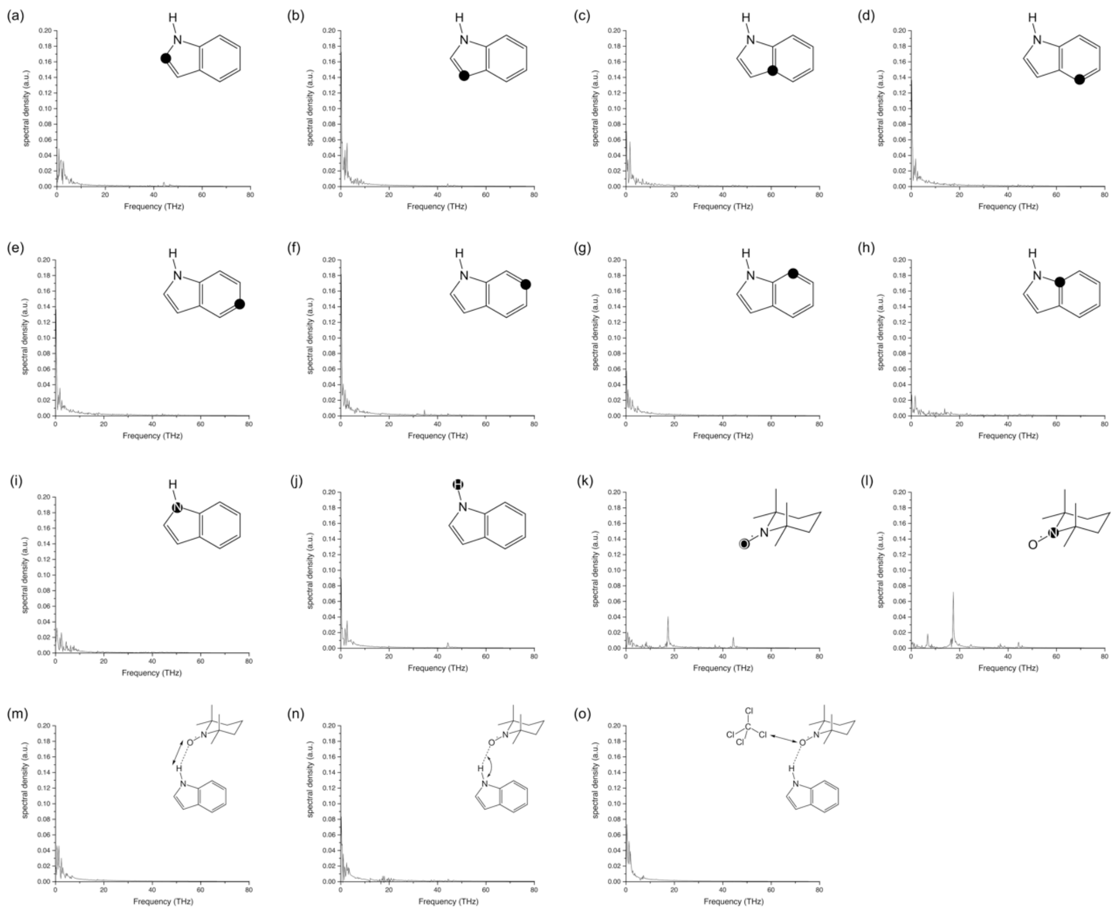

Supplementary Figure 7. Spectral density function $j(\omega)$ of spin density fluctuation (a-l) and the intermolecular dynamics (m-o) in indole-TEMPO complex. All these spectral density functions are obtained by the Fourier transform of the corresponding normalized autocorrelation functions. The spectral density functions of TEMPO NO moiety show coherent process(es) in the IR frequency range. All the spectral density functions feature the initial decay together with oscillations in the low THz regime. The low THz region of the spectral density functions is shown in the next session of Supplementary Figure 5 on the following page.

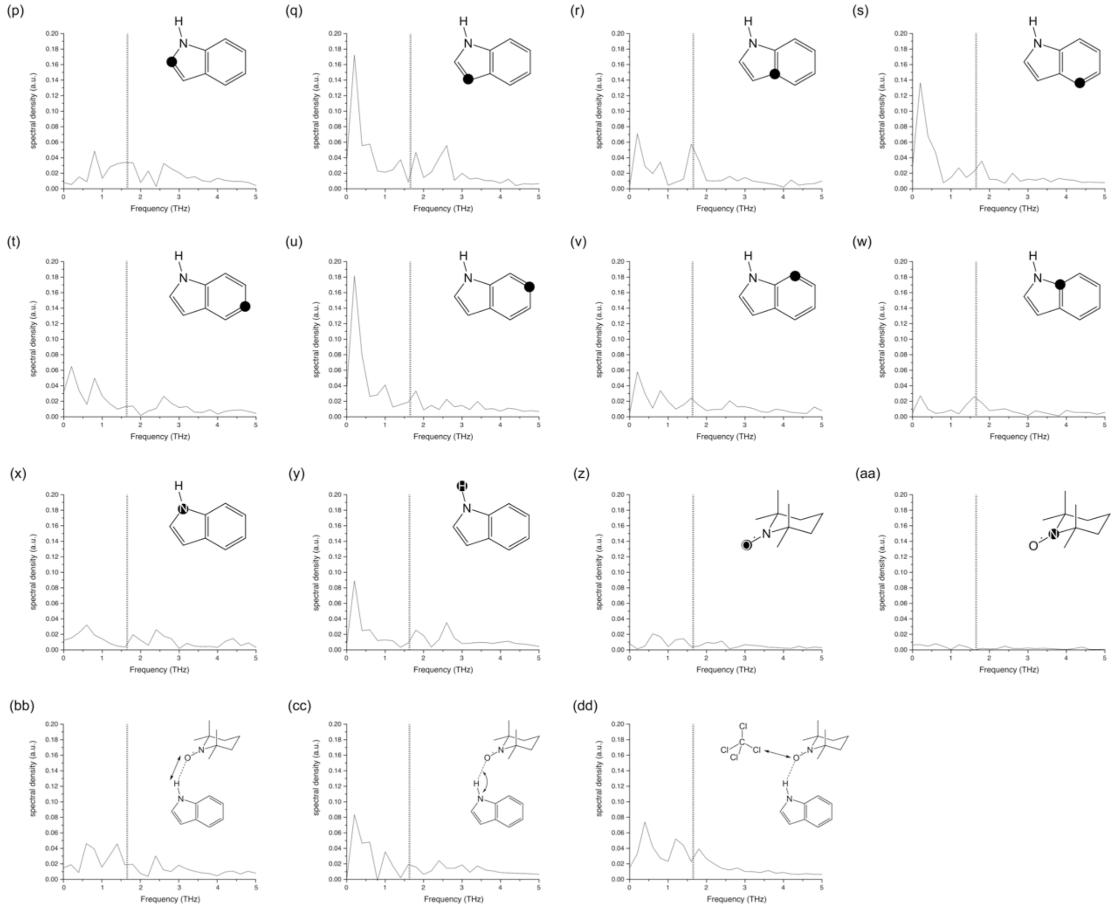

Supplementary Figure 7. (continue) Low THz region (p-dd) of the spectral density function shown in (a-o). The dashed line shows the position of e-^{13}C ZQ transition frequency at 9.4 T.

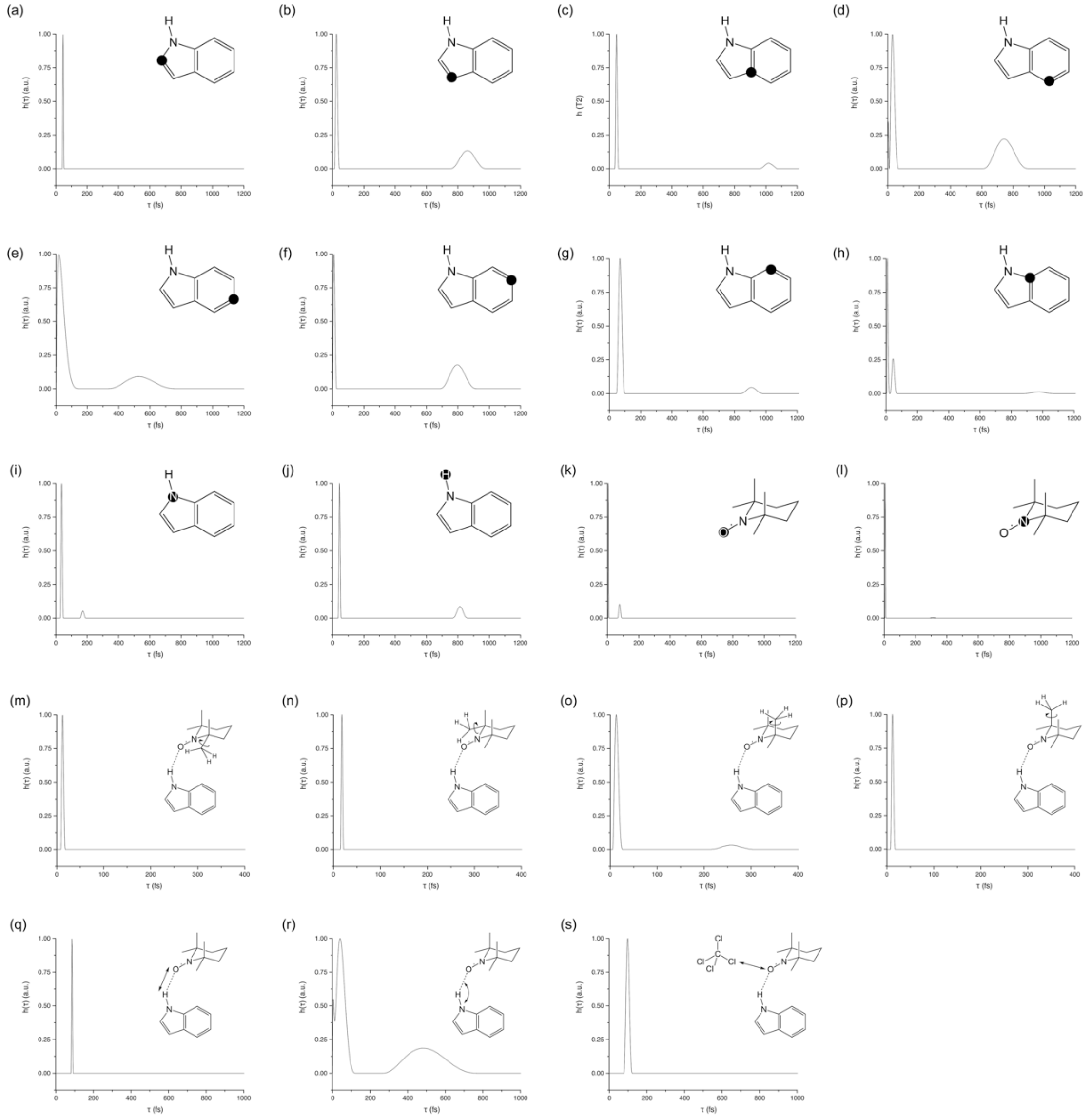

Supplementary Figure 8. Decay time constant of autocorrelation functions calculated by the inverse Laplace transform using CORTIN[17]. This protocol fits the decay of autocorrelation function as a distribution of decay time constants following the highly simplified exponential decay model. Therefore the results here only present the semi-quantitative description of the decay of autocorrelation function due to the random process. All results are normalized for clarity.

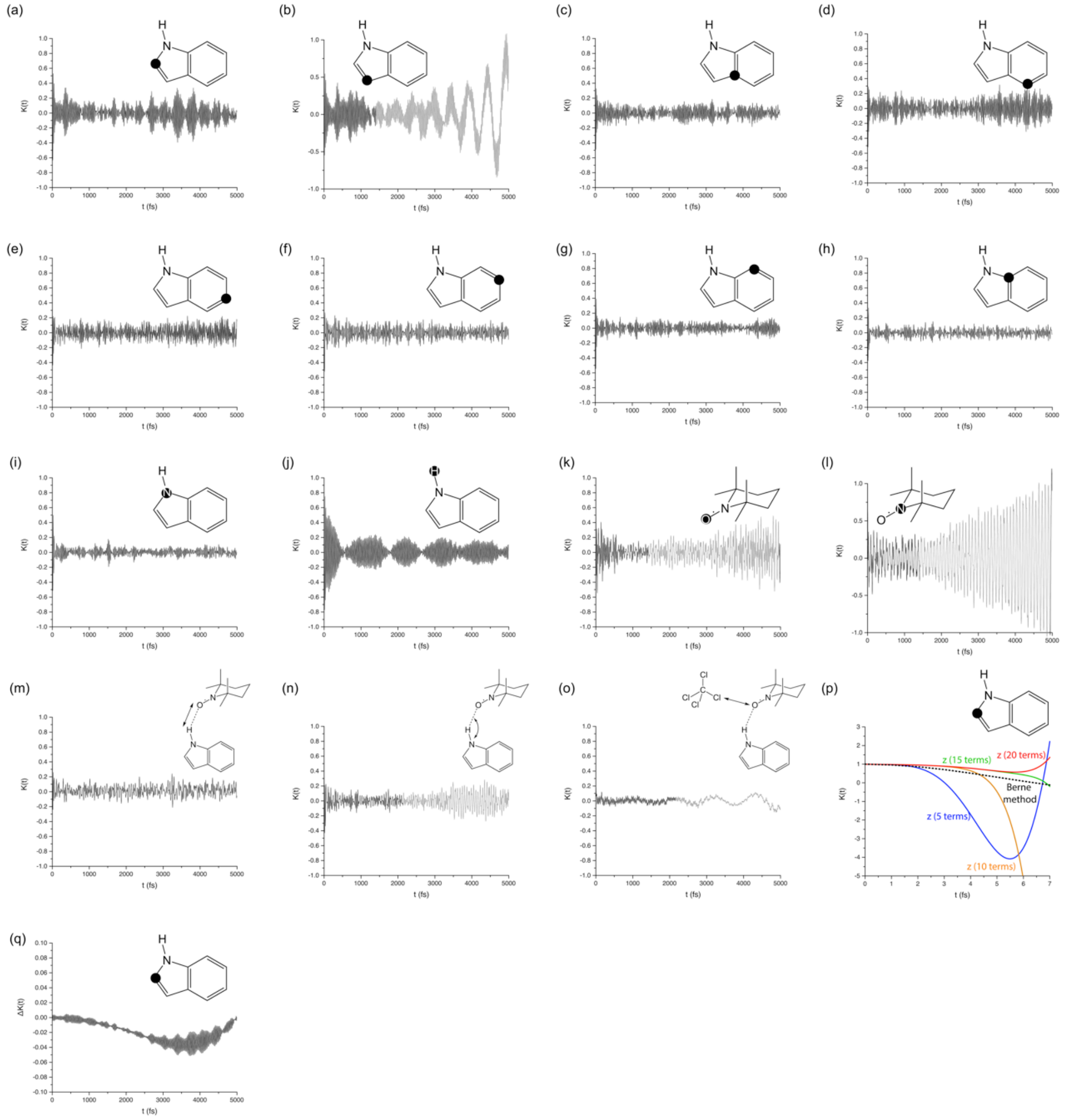

Supplementary Figure 9. Memory functions $K(t)$ of spin density fluctuations (a-k) and intermolecular dynamics (m-o) in indole-TEMPO complex. All memory functions are calculated using a numerical approach described by Berne et al.[18]. More details about the computation of memory function can be found in **S5**. The decay of $K(t)$ presents the length of the memory effect. The oscillation components are originated from the oscillations of corresponding $G(t)$ or $\rho(t)$, which are better visualized in $K(t)$. The truncated Z-transform was also use for calculating $K(t)$. However, as shown in (p), all levels of truncation (colored curves) lead to the significant deviation from the proper numerical solution (dashed black curve) and even to the rapid divergence in a few fs. Memory functions $K(t)$ can also be computed using an alternative numerical approach as described in **S5**. The deviation between two numerical approaches is shown in (q). The differences between these two numerical solutions escalate slowly and remain at a small scale. For some specific autocorrelation function $G(t)$, the numerical approach still leads to the observable divergence after more than 1000 steps (b,k,l,n.o, gray part). Nevertheless such divergence does not mask the fundamental features of those memory functions.

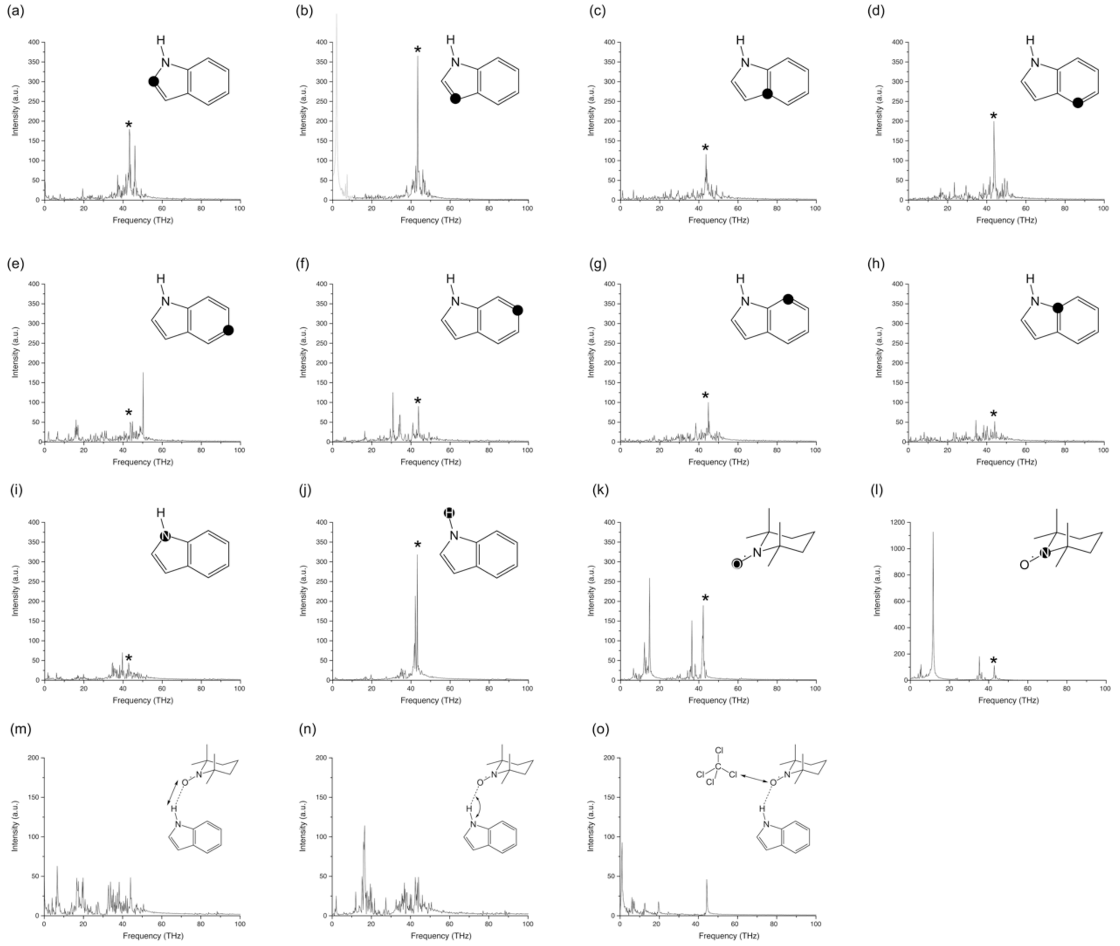

Supplementary Figure 10. Fourier transformed amplitude spectrum of memory functions $K(t)$ of the spin density fluctuation (a-l) or intermolecular dynamics (m-o) in indole-TEMPO complex. The presence of memory effect is indicated by the non-zero values at the zero frequency. The peaks in these spectra shows the presence of coherent dynamics in the original trajectory $\rho(t)$. The * symbol in (a-l) suggest a shared coherent component in the IR frequency range within the fluctuations of spin density within the whole complex, which suggests certain IR modes could modulate the electronic structure of the whole complex.

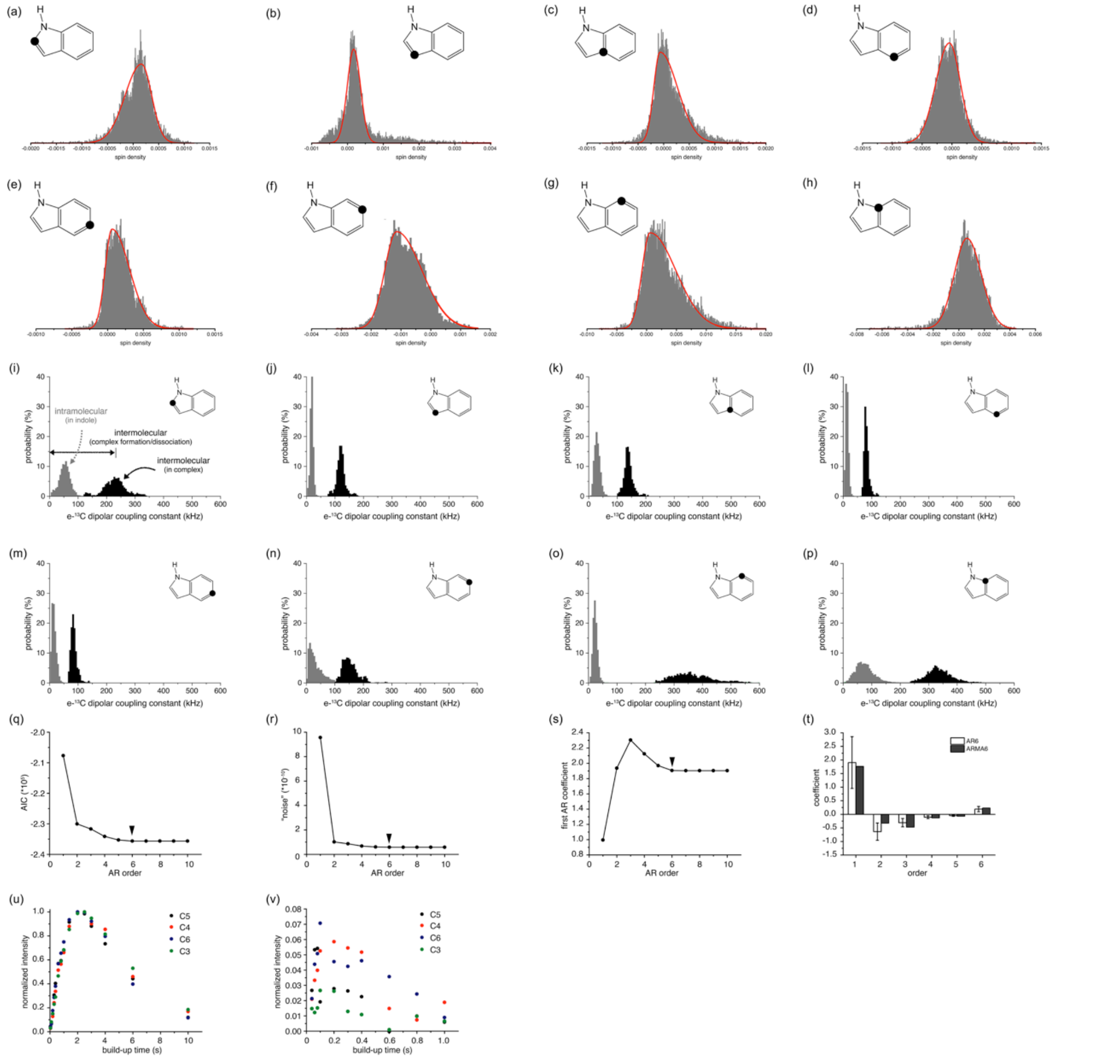

Supplementary Figure 11. Mechanistic complexity of ODNP in TEMPO-indole complex. (a-h) Most carbon spin density distribution in indole shows asymmetric distribution with significantly crossing of zero-spin density condition. (i-p) Beside the intermolecular dipolar coupling, the intramolecular dipolar coupling originated from the spin density on other indole carbons also could contribute to the dipolar ODNP. (q-s) The spin-density trajectory of indole C2 was fitted using AR(p) models of different order *p*. The fitted parameters, namely AIC (indicator of fitting quality), the noise level in the model and the scale of the first (main) component, show that the fitting quality does not improve beyond the *p* order 6. Therefore, the trajectory shows the "memory" of at least 6 steps (6 fs). (t) The trajectory was also fitting using a more complicated ARMA model, which shows that two models converge at higher orders (p ≥ 6). The errors presented in panel (t) are from the model fitting directly. (u-v). ^{1}H-^{13}C hNOE build-up kinetics of indole (2 M) in the absence (u) and presence (v) of TEMPO (100 mM) in CCl_4 solvent. All these kinetics were extracted from on a series of 2D ^{1}H-^{13}C HOESY spectra. The maximum signal intensity of each indole carbon in the absence of TEMPO (u) was normalized. The signal intensities in the presence of TEMPO (v) were scaled according to the normalized diamagnetic signal intensity.

Supplementary References

1 Abragam, A. *The principles of nuclear magnetism*. (Clarendon Press, Oxford, 1961).

2 Bertini, I., Luchinat, C. & Parigi, G. *Solution NMR of Paramagnetic Molecules : Applications to Metallobiomolecules and Models*. (Elsevier Science Ltd., Amsterdam, 2001).

3 Noack, F., Krüger, G. J., Müller-Warmuth, W. & Van Steenwinkel, R. Stochastische Prozesse in Spinsystemen. *Z. Naturforsch. A* **22**, 2102-2108 (1967).

4 Müller-Warmuth, W., Van Steenwinkel, R. & Noack, F. Dynamic nuclear polarization experiments on 19F in solutions and their interpretation by the 'pulse model' of molecular collisions. *Z. Naturforsch. A* **23**, 506-513 (1968).

5 Gladchenko, L. F. & Pikulik, L. G. Dipole moment of the excited state in indole and tryptophan. *Zh. Prikl. Spektrosk.* **6**, 355-360 (1967).

6 Terazima, M., Tenma, S., Watanabe, H. & Tominaga, T. Translational diffusion of chemically stable and reactive radicals in solution. *J. Chem. Soc., Faraday Trans.* **92**, 3057-3062 (1996).

7 Qiu, Z. W., Grant, D. M. & Pugmire, R. J. Paramagnetic C-13 shifts induced by the free-radical Tempo .2. nitrogen-heterocycles. *J. Am. Chem. Soc.* **106**, 557-563 (1984).

8 Liu, G. Q. et al. One-thousand-fold enhancement of high field liquid nuclear magnetic resonance signals at room temperature. *Nat. Chem.* **9**, 676-680 (2017).

9 Iiyama, T. et al. Molecular assembly structure of CCl4 in graphitic nanospaces. *J. Phys. Chem. B* **101**, 3037-3042 (1997).

10 Merunka, D. & Peric, M. Measuring radical diffusion in viscous liquids by electron paramagnetic resonance. *J. Mol. Liq.* **277**, 886-894 (2019).

11 Orlando, T. et al. Dynamic nuclear polarization of C-13 nuclei in the liquid state over a 10 Tesla field range. *Angew. Chem. Int. Ed.* **58**, 1402-1406 (2019).

12 Cryer, J. D. & Chan, K. S. *Time Series Analysis: With Applications in R, second edition*. (Springer-Verlag, New York, 2008).

13 Cavallotti, C., Metrangolo, P., Meyer, F., Recupero, F. & Resnati, G. Binding energies and F-19 nuclear magnetic deshielding in paramagnetic halogen-bonded complexes of TEMPO with haloperfluorocarbons. *J. Phys. Chem. A* **112**, 9911-9918 (2008).

14 Kucuk, S. E., Biktagirov, T. & Sezer, D. Carbon and proton Overhauser DNP from MD simulations and ab initio calculations: TEMPOL in acetone. *Phys. Chem. Chem. Phys.* **17**, 24874-24884 (2015).

15 Daube, D. et al. Heteronuclear cross-relaxation under solid-state dynamic nuclear polarization. *J. Am. Chem. Soc.* **138**, 16572-16575 (2016).

16 Hoffmann, M. M. et al. Directly vs indirectly enhanced C-13 in dynamic nuclear polarization magic angle spinning NMR experiments of nonionic surfactant systems. *J. Phys. Chem. C* **121**, 2418-2427 (2017).

17 Provencher, S. W. CONTIN: A general purpose constrained regularization program for inverting noisy linear algebraic and integral equations. *Comput. Phys. Commun.* **27**, 229-242 (1982).

18 Berne, B. J. & Harp, G. D. On the Calculation of Time Correlation Functions in *Advances in Chemical Physics*, 63-227 (John Wiley & Sons Ltd., New York, 1970).
